# Development and validation of a phospholipid metabolism-associated lncRNA model for prognostic stratification and therapeutic guidance in HNSCC

**DOI:** 10.1186/s12957-026-04299-2

**Published:** 2026-03-12

**Authors:** Yang Zhang, Shuanggong Liu, Guangxu Song, Kewen Wang, Zhi Zhou, Yueying Xiao, Dongjin Wu

**Affiliations:** 1 Department of Orthopaedics, The Second Qilu Hospital of Shandong University, 247 Beiyuan Street, Jinan, Shandong 250033 People’s Republic of China; 2 Department of Orthopedics, College of Medicine, Zhongda Hospital, Southeast University, 86 Chongwen Street, Nanjing, Jiangsu 210009 People’s Republic of China

**Keywords:** HNSCC, Phospholipid metabolism, Long non-coding RNA, Prognostic risk model

## Abstract

Alterations in phospholipid metabolism are increasingly recognized as critical in cancer progression, yet their specific contributions to head and neck squamous cell carcinoma (HNSCC) remain unclear. This study aimed to establish a prognostic model based on phospholipid metabolism-related long non-coding RNAs (lncRNAs) to predict clinical outcomes in HNSCC patients. Transcriptomic data from The Cancer Genome Atlas (TCGA) were analyzed to identify differentially expressed lncRNAs linked to phospholipid metabolism. Using univariate and multivariate Cox regression analyses, a 10-lncRNA signature (AC104041.1, AC106820.3, AL355488.1, LINC01305, AC133644.1, AC091185.1, AL158166.1, AL512274.1, DBH-AS1, AC025176.1) was developed. The model’s predictive accuracy was confirmed through Kaplan–Meier survival analysis, time-dependent receiver operating characteristic (ROC) curves, and decision curve analysis (DCA), surpassing traditional clinical indicators for overall survival (OS). Gene set enrichment analysis, immune profiling, and drug sensitivity testing revealed distinct biological differences between clusters. Low-risk patients and those in cluster 2 exhibited better prognoses, enhanced immune infiltration, and heightened immune activity, while high-risk patients in cluster 1 showed increased sensitivity to chemotherapeutic agents. Functional assays demonstrated that AL158166.1 knockdown inhibited HNSCC cell proliferation, migration, invasion, and phospholipid metabolism, likely through suppression of the PI3K/AKT/mTOR pathway. These findings suggest that AL158166.1 plays a pivotal role in HNSCC progression. In conclusion, the proposed lncRNA-based prognostic model provides a reliable tool for survival prediction and personalized treatment guidance in HNSCC, highlighting promising therapeutic targets and advancing precision immunotherapy and chemotherapy strategies.

**Graphical Abstract:**

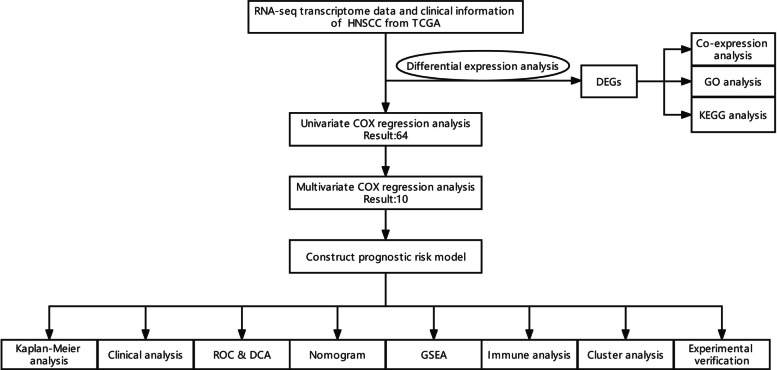

**Supplementary Information:**

The online version contains supplementary material available at 10.1186/s12957-026-04299-2.

## Highlights

• This study established a 10-lncRNA signature related to phospholipid metabolism for predicting HNSCC prognosis.

• The model demonstrated superior predictive accuracy over traditional clinical indicators in overall survival analysis.

• Immune profiling and drug sensitivity analysis revealed distinct biological differences between risk clusters.

• AL158166.1 knockdown inhibited HNSCC progression by suppressing the PI3K/AKT/mTOR pathway.

**Supplementary Information:**

The online version contains supplementary material available at 10.1186/s12957-026-04299-2.

## Introduction

Head and neck squamous cell carcinoma (HNSCC) originates from the mucosal epithelium located in areas including the oral cavity, oropharynx, larynx, nasopharynx, and hypopharynx, making it the most prevalent malignancy in the head and neck region [1]. Globally, it ranks among the top ten most commonly diagnosed cancers, with approximately 930,000 new cases and 460,000 related deaths reported in 2020 [2]. Despite ongoing advancements in surgical techniques, chemotherapy, radiotherapy, and immunotherapy, the five-year overall survival rate for patients with HNSCC remains unsatisfactory [3–5]. The delayed diagnosis of HNSCC, frequently occurring at advanced stages, is primarily due to the absence of effective clinical screening markers [6]. Moreover, conventional staging methods that rely on pathological features are insufficient for precisely predicting the prognosis of patients with HNSCC [7]. Consequently, there is an urgent need to identify effective biomarkers to facilitate the early detection, accurate diagnosis, and prognostic evaluation of HNSCC.

Dysregulated cellular metabolism is closely associated with tumor initiation and progression [8, 9]. Phospholipid metabolism is an essential component of lipid metabolism, performing multiple cellular functions, including chemical energy storage, regulation of cell signaling, preservation of cell membrane integrity, and support of intercellular interactions within tissues [10]. Altered phospholipid metabolism is a hallmark feature of most cancers [11, 12]. Previous studies have suggested links between phospholipid metabolism and intestinal tumorigenesis [13, 14]. Phospholipase A2 (PLA2), a key enzyme in phospholipid metabolism, promotes tumorigenesis and enhances cancer aggressiveness by interacting with Annexin A1 [15]. Earlier studies have similarly shown a significant increase in lipid-derived phospholipid concentrations in the HNSCC tumor tissue compared to healthy controls [16]. Recent studies have further highlighted the critical role of the tumor immune microenvironment and metabolic regulation in the progression of head and neck squamous cell carcinoma. A tertiary lymphoid structures–related gene signature has been shown to effectively predict prognosis and immune cell infiltration patterns in HNSCC, underscoring the importance of immune-associated molecular features in patient stratification [17]. In addition, SLC2A3, a metabolism-related gene, has been reported to promote HNSCC development through modulation of the tumor microenvironment, further linking metabolic reprogramming to immune regulation and disease progression [18]. These findings collectively suggest that metabolism-associated molecular signatures are closely intertwined with immune characteristics and clinical outcomes in HNSCC.

LncRNAs represent a heterogeneous class of transcripts that are longer than 200 nucleotides [19]. LncRNAs function as emerging regulators that influence gene expression across a wide array of both normal and disease-associated biological processes [20–22]. Mounting evidence underscores the intricate and precise regulatory roles of lncRNAs in the initiation and advancement of cancer, where they function as either oncogenes or tumor suppressors [23, 24]. Their regulatory influence spans not only the modulation of cancer cell proliferation, differentiation, invasion, and metastasis but also includes the orchestration of metabolic reprogramming within cancer cells [25, 26]. Previous studies have suggested lncROPM can regulate PLA2G16‑mediated phospholipid metabolism to promote breast cancer stem cells (BCSCs) [27]. LncRNAs are emerging as potential candidates for cancer diagnosis, prognostic evaluation, and therapy, owing to their widespread expression across the genome and their tissue-specific expression patterns in various body tissues [28, 29]. To the best of our knowledge, existing studies on phospholipid metabolism-related lncRNAs have largely focused on individual molecules or specific cancer types, rather than providing a systematic and disease-specific evaluation in HNSCC. Current lncRNA-based prognostic models for HNSCC rarely integrate phospholipid metabolic features, and their associations with the tumor immune microenvironment and therapeutic responsiveness remain insufficiently characterized. Furthermore, most bioinformatically identified lncRNAs lack experimental validation to support their biological relevance and mechanistic involvement in phospholipid metabolism within HNSCC. Therefore, an integrative prognostic framework that incorporates phospholipid metabolism-associated lncRNAs, immune characteristics, therapeutic implications, and functional validation is still lacking.

The objective of this study was to develop a predictive model for assessing the risk in HNSCC patients by utilizing pairs of lncRNAs associated with phospholipid metabolism. Initially, we identified phospholipid metabolism-related lncRNAs with significant survival relevance through RNA-seq data from HNSCC patients. Multiple algorithms were employed to construct the model. We then evaluated the predictive performance of the risk score and performed additional analyses, including Gene Set Enrichment Analysis (GSEA), immune microenvironment characterization, and drug responsiveness across different patient groups. The samples were subsequently divided into Cluster 1 and Cluster 2 based on the expression levels of risk-associated lncRNAs, followed by a comparative analysis with the earlier findings. Lastly, we validated our results through various experimental approaches. Our findings indicate that silencing AL158166.1 notably suppressed the migratory, invasive, and proliferative capacities of HNSCC cells. Mechanistically, AL158166.1 may enhance the malignant phenotype of HNSCC cells through the activation of the PI3K/AKT/mTOR pathway. In conclusion, our model demonstrates robust prognostic potential for HNSCC and underscores the potential for advancing immunotherapy strategies and optimizing clinical therapeutic selections.

## Materials and methods

### Data collection

The RNA-seq and clinical data for HNSCC samples were obtained from TCGA (https://portal.gdc.cancer.gov/). Detailed clinical characteristics of the patients are provided in Table 1. We selected the phospholipid metabolism-related gene set (M649) from the Molecular Signatures Database v7.5.1 (https://www.gsea-msigdb.org/gsea/msigdb/), which was subsequently incorporated into our analysis.

**Table 1:** The clinical characteristics of patients with HNSCC (*n* = 527)

Characteristics		No
Age	≤ 65	345
	> 65	182
Gender	Female	142
	Male	385
Grade	Grade1	63
	Grade2	310
	Grade3	125
	Grade4	7
	Unknow	22
Stage	Stage I	27
	Stage II	73
	Stage III	82
	Stage IV	270
	Unknow	75
T stage	T0	1
	T1	49
	T2	139
	T3	101
	T4	175
	Unknow	62
N stage	N0	179
	N1	68
	N2	172
	N3	8
	Unknow	100
M stage	M0	190
	M1	1
	Unknow	336

### Differential expression analysis and functional enrichment analysis

To explore phospholipid metabolism–related lncRNAs, a weighted gene co-expression network analysis (WGCNA)–based strategy was applied using the “WGCNA” R package. An unsigned co-expression network was constructed based on normalized expression data. The soft-thresholding power (β) was selected according to the scale-free topology criterion. Specifically, the lowest β value at which the scale-free topology fit index exceeded 0.85 was chosen while maintaining adequate mean connectivity of the network. Based on this criterion, a soft-thresholding power of β = 8 was used for network construction. The resulting adjacency matrix was transformed into a topological overlap matrix (TOM).Based on the constructed co-expression network, lncRNAs significantly correlated with phospholipid metabolism–related genes were identified using Pearson correlation analysis (|cor|> 0.4, *p* < 0.001). ifferential expression of phospholipid metabolism–related genes and lncRNAs between HNSCC and normal tissues was determined using the limma package in R software, applying the criteria of |logFC|> 1 and a false discovery rate (FDR) of < 0.05. ene Ontology (GO) analysis, encompassing molecular function (MF), biological process (BP), and cellular components (CC), was performed using the ClusterProfiler package. Additionally, the same approach was employed for Kyoto Encyclopedia of Genes and Genomes (KEGG) pathway analysis. Significance was considered for FDR and p-values < 0.05 in the enrichment analyses.

### Construction of risk assessment model

We performed univariate Cox regression analysis to evaluate the prognostic implications of lncRNAs associated with phospholipid metabolism. Following this, we utilized multivariate Cox regression analysis to construct a prognostic risk model with the glmnet R package. The calculation of risk scores was executed using the following formula:

$$\text{risk score}={\sum }_{k=1}^{n}coef\left({lncRNA}^{k}\right)*expr({lncRNA}^{k})$$

Here, "Coef" represents the multivariate Cox regression model coefficient for the respective lncRNA. We segregated HNSCC patients into high-risk and low-risk groups based on the median risk score.

### Evaluation of the risk model

Kaplan–Meier survival analysis was utilized to identify differences between the two groups. To assess the model's potential as an independent clinical prognostic factor, we performed both univariate and multivariate Cox regression analyses, incorporating the risk score alongside clinicopathological variables. The sensitivity and specificity of the prognostic signatures for HNSCC, relative to other clinicopathological factors, were evaluated through ROC and DCA. A nomogram was constructed, integrating the prognostic signatures, to predict the 1-, 2-, and 3-year overall survival (OS) of HNSCC patients. Additionally, the relationship between phospholipid metabolism-related lncRNAs and clinicopathological characteristics was analyzed using logistic regression, and a heatmap was generated to visualize these associations. Subsequently, patients in each dataset were stratified into high-risk and low-risk groups according to the cohort-specific median risk score. Kaplan–Meier survival curves were generated to compare overall survival between the two groups. Time-dependent receiver operating characteristic (ROC) curves were constructed to evaluate the sensitivity and specificity of the prognostic model in the TCGA training cohort and the GSE41613 validation cohort. Finally, the expression patterns of the signature genes, along with the distributions of survival status and risk scores, were visualized in both the TCGA and GEO cohorts.

### GSEA

Using the curated gene set (kegg.v7.4.symbols.gmt), we conducted Gene Set Enrichment Analysis (GSEA) via the GSEA software (https://www.gsea-msigdb.org/gsea/login.jsp) to identify pathways that were significantly enriched between the low-risk and high-risk groups. The analysis was performed with a threshold of *p*-value < 0.05 and false discovery rate (FDR) < 0.25.

### The investigation of the tumor immune microenvironment (TIME) and immune checkpoints

To assess the correlation between the risk score and the presence of immune infiltrating cells, we utilized established methodologies for quantifying immune infiltration levels in HNSCC patients using TIMER2.0 (http://timer.comp-genomics.org/). These methodologies included TIMER, CIBERSORT, CIBERSORT-ABS, QUANTISEQ, MCPcounter, XCELL, and EPIC (Supplementary Table 1). Additionally, we conducted comparisons of immune function and the activation of immune checkpoints between the low-risk and high-risk groups using the ggpubr R package.

### Drug sensitivity analysis

To investigate differences in drug sensitivity between the two groups, we calculated the half-maximal inhibitory concentration (IC50) for predictive purposes using the pRRophetic package. Statistical significance was determined with a threshold of *P* < 0.05.

### Clusters based on prognostic lncRNAs

We conducted an analysis of potential molecular subgroups that may respond to immunotherapy using the ConsensusClusterPlus (CC) package, based on the expression of prognostic phospholipid metabolism-related lncRNAs. Principal component analysis (PCA), T-distributed stochastic neighbor embedding (t-SNE), and Kaplan–Meier survival analyses were performed using the Rtsne R package. Additionally, immune analyses and drug sensitivity comparisons were carried out using the GSVA Base and pRRophetic R packages.

### Cell lines and transfection

The human HNSCC cells, FaDu (CL-0083, Pricella, China), and SCC-9 (CL-0571, Pricella, China), were cultured in minimum essential medium (Pricella, China) with 10% fetal bovine serum (ViviCell, Shanghai, China) and 1% penicillin/streptomycin antibiotics at 37 °C, 5%CO_2_. Small interfering RNA (siRNA) targeting AL158166.1 (referred to as si-AL158166.1) and a corresponding negative control (si-NC) were obtained from Gene Pharma in Shanghai, China. FaDu and SCC-9 cells were seeded in 6-well plates at a density of 1 × 106 cells per well and then transfected with either si-AL158166.1 or si-NC using X-tremeGENE siRNA transfection reagent from Roche, Mannheim, Germany, following the manufacturer's provided instructions.The following siRNA sequences were used in this study:si-AL158166.1–1, sense, 5′-GUGGCAAGCUACCCUAUAUTT-3′; antisense: 5′-AUAUAGGGUAGCUUGCCACTT-3′; si-AL158166.1–2, sense, 5′-GCUUCCUCUAUUGUUAAAUTT-3′, antisense, 5′-AUUUAACAAUAGAGGAAGCTT-3′; si-AL158166.1–3, sense, 5′-GCUCACUGAACAGGAAUUATT-3′, antisense: 5′-UAAUUCCUGUUCAGUGAGCTT-3′.

### Quantitative real-time PCR (qRT-PCR)

Total RNA was isolated from transfected FaDu and SCC-9 cells using TRIzol (Invitrogen, USA). Subsequently, cDNA synthesis was carried out with the HyperScript III 1 st Strand cDNA Synthesis Kit (EnzyArtisan, China). Amplification reactions for qRT-PCR were conducted using Power SYBR Green PCR Master Mix (Applied Biosystems, USA) and 7500 Fast real-time PCR system (ThermoFisher, USA). The relative expression of genes was determined using the 2 − ΔΔCt method, with transcript levels normalized to those of GAPDH. The primer sequences employed in this study were as follows: AL158166.1, forward, 5′-AAAGAGGGTGCCTCAACCG-3′, reverse, 5′-AACCTCCCTCTTTGGCAACTTT-3′; GAPDH, forward, 5′-TGGAAATCCCATCACCATCT-3′, reverse, 5′-TGGACTCCACGACGTACTCA-3′.

### Cell invasion assay

Cell proliferation was evaluated utilizing the Cell Counting Kit-8 (CCK-8; Beyotime, Beijing, China) assay. FaDu and SCC-9 cells were seeded in 96-well plates at a density of 5 × 103 cells per well and incubated at 37 °C for 0, 1, 2, 3, and 4 days. At designated time points, 10 µl of CCK-8 reagent was added to each well, followed by measuring the optical density (OD value) at 450 nm using a microplate reader (Bio-Rad, USA). Cell proliferation was evaluated using the EdU-555 Cell Proliferation Detection Kit (Epizyme, China). Following treatment, cells were incubated with the EdU reagent for 2 h, fixed with 4% paraformaldehyde, and permeabilized with 0.5% Triton X-100. Nuclei were then stained with Hoechst. In the colony formation experiments, we introduced 600 transfected FaDu and SCC-9 cells into each well of 6-well plates. After a period of fourteen days, the count of colonies was recorded. This procedure was carried out in triplicate for each assay.

### Wound healing assay

FaDu and SCC-9 cells were grown until they formed a confluent monolayer in 6-well culture plates. On the day of the experiment, a wound was deliberately introduced across the well using a sterile pipet tip. The cells were then softly washed with PBS and the culture medium was replenished. Photomicrographs were captured with a phase-contrast light microscope at both the 0-h and 24-h time points. Replicates of all experiments were conducted in triplicate.

### Transwell assays

In brief, Transwell chambers (Greiner Bio-One, Austria) were prepared by adding Matrigel (30 µg/well) (Becton Dickinson, USA). Subsequently, the upper chamber received 5 × 104 FaDu or SCC-9 cells in serum-free medium, while the lower chamber contained serum-containing medium. Following a 48-h incubation, the migrated cells were fixed with 4% paraformaldehyde and subsequently stained using crystal violet.

### Protein extraction and western blot

Western blot analysis was employed to evaluate the expression of pertinent proteins. Cells were lysed for total protein extraction using RIPA Lysis Buffer (obtained from Beyotime, Beijing, China) supplemented with protease and phosphatase inhibitors. The protein concentration was determined using a BCA assay kit (also sourced from Beyotime, Beijing, China). Subsequently, 15 μg of total proteins from each experimental group were loaded onto either 10% or 12% sodium dodecyl sulfate–polyacrylamide gel electrophoresis (SDS-PAGE) gels for separation. Following electrophoresis, these proteins were transferred onto polyvinylidene difluoride (PVDF) membranes. The membranes were blocked using a TBST solution containing 5% nonfat dried milk and then incubated with primary antibodies overnight at 4 °C. Primary antibodies against E-cadherin (1:1000, CST, USA),N-cadherin (1:1000, CST, USA), Vimentin (1:1000, Affinity, China), STAT3 (1:1000, Affinity, China), CHK-α (1:1000, CST, USA), FASN (1:1000, Affinity, China), PLD3 (1:1000, Affinity, China), PLD1 (1:1000, Affinity, China), T-PI3K (1:1000, CST, USA),p-PI3K (1:1000, CST, USA), T-AKT (1:1000, CST, USA), p-AKT (1:1000, CST, USA), T-mTOR (1:1000, CST, USA), p-mTOR (1:1000, CST, USA), β-actin (1:1000, Affinity, China), were used in this study. Following three rounds of membrane washing, the membranes were exposed to a 1-h incubation at room temperature with the respective secondary antibodies. Protein bands were subsequently detected using an enhanced ECL kit (Millipore, USA) in combination with a chemiluminescence imaging system (Tanon-4800, Shanghai, China). Subsequently, the data were analyzed using ImageJ software (National Institutes of Health, USA).

### Immunofluorescence

Cells were plated onto 20-mm confocal dishes and cultured in DMEM/F12 without serum for 24 h after reaching approximately 80% confluence. Based on their respective experimental groups, the cells were then subjected to specific treatments. After being washed three times with PBS, the cells were fixed in 4% paraformaldehyde for 15 min, followed by permeabilization with 0.3% Triton X-100 in PBS for another 15 min. The cells were washed again with PBS and subsequently blocked with 10% bovine serum albumin (BSA) for 1 h at room temperature. Primary antibodies were applied, and the cells were incubated overnight at 4 °C. After three additional PBS washes, the cells were treated with a fluorescein isothiocyanate (FITC)-conjugated secondary antibody in the dark for 1 h at room temperature. Nuclear staining was performed with DAPI for 5 min in darkness, and the samples were mounted using an anti-fade mounting medium. Immunofluorescence images were captured using a laser scanning confocal microscope (Zeiss LSM800, Germany), and fluorescence intensity was quantified using ImageJ software, with normalization to the DAPI signal.

### Statistics analysis

Experimental data underwent analysis using R software (version 4.1.0) and GraphPad Prism 8.0 (GraphPad Software Inc., USA). Results are expressed as the mean ± standard deviation (SD). Cox regression was employed for both univariate and multivariate overall survival analysis. Spearman correlation analysis was employed to evaluate the correlation coefficient between the risk score and scores of immune infiltration cells. Student's t-test was utilized for the comparison of two groups, whereas one-way ANOVA followed by Tukey’s post hoc test was applied for comparisons across multiple groups. Statistically significant differences were denoted at *P* < 0.05, with *P < 0.05, **P < 0.01, and ***P < 0.001 indicating significance levels.

## Results

### Functional enrichment analysis

**Fig. 1:**
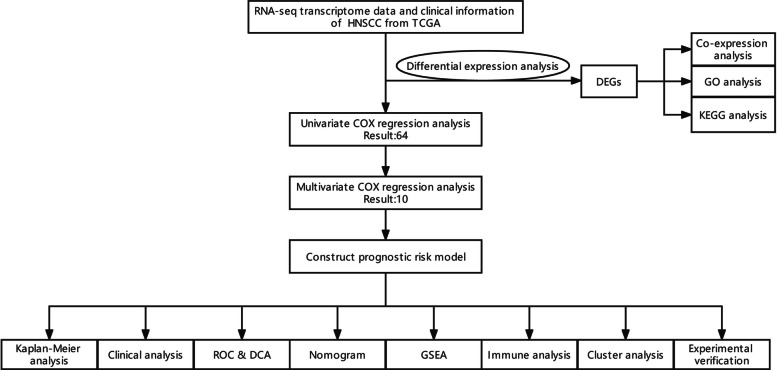
Flow diagram of study design

**Fig. 2:**
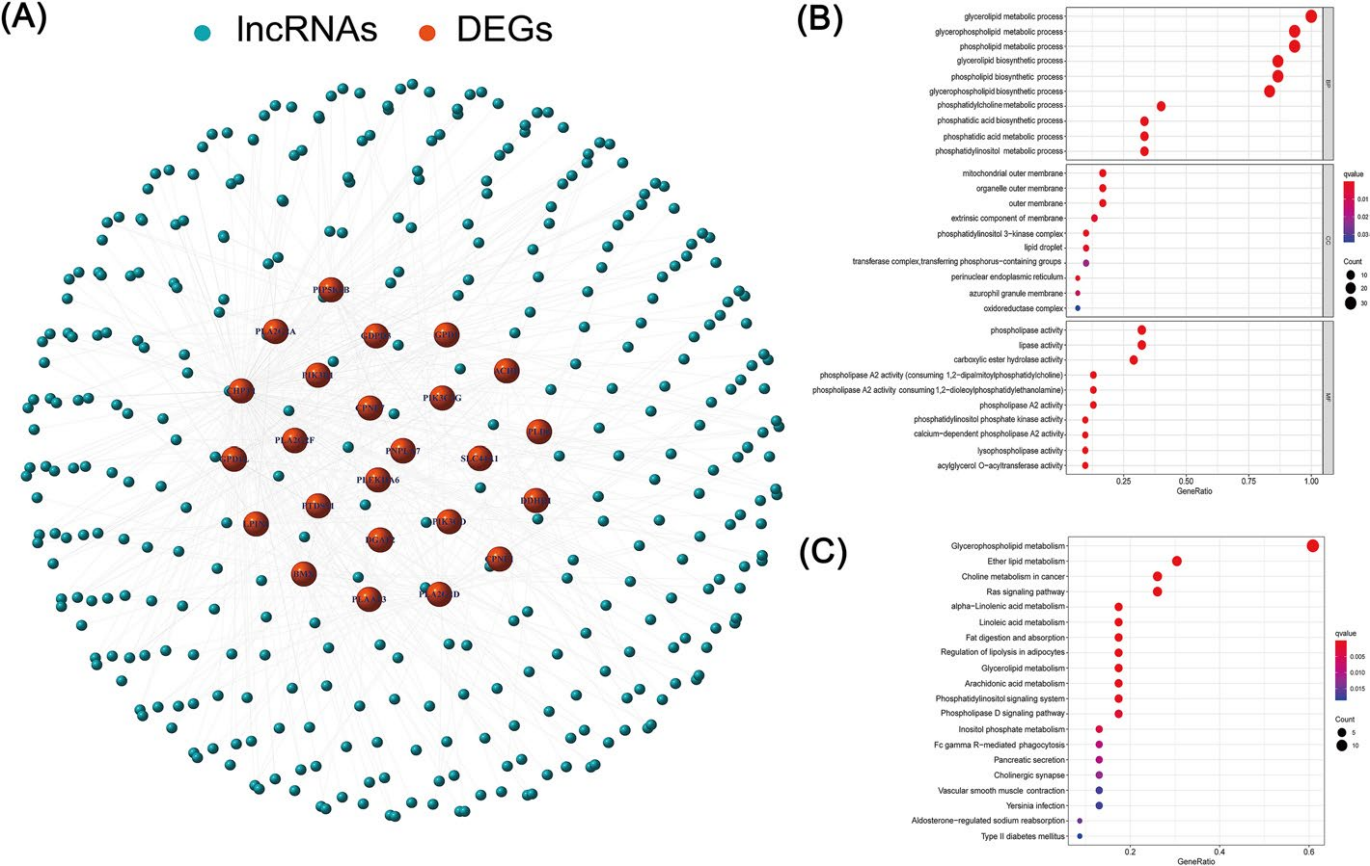
Differential expression analysis and functional enrichment analysis. **A** Network between phospholipid metabolism-related DEGs and lncRNAs. **B** GO analysis. **C** KEGG analysis

The study's workflow is depicted in Fig. 1. We identified 31 differentially expressed genes (DEGs) associated with phospholipid metabolism, consisting of 12 upregulated and 19 downregulated genes. The interaction network between phospholipid metabolism-related DEGs and lncRNAs is presented in Fig. 2A. To explore the potential functions of these DEGs, we conducted GO and KEGG pathway analyses. Results from the biological process analysis indicated that the 31 DEGs are significantly associated with glycerophospholipid metabolic processes, phospholipid metabolic processes, phospholipid biosynthetic processes, and phosphatidylcholine metabolic processes. In the cellular component analysis, these DEGs were primarily localized to the mitochondrial outer membrane, organelle outer membrane, phosphatidylinositol 3-kinase complex, and lipid droplets. Molecular function analysis showed significant enrichment in phospholipase activity, carboxylic ester hydrolase activity, phospholipase A2 activity, and phosphatidylinositol phosphate kinase activity. KEGG pathway analysis revealed that these genes are predominantly involved in glycerophospholipid metabolism, choline metabolism in cancer, the Ras signaling pathway, the phosphatidylinositol signaling system, the phospholipase D signaling pathway, and inositol phosphate metabolism (Fig. 2B-C).

### Construction of the model based on phospholipid metabolism-related lncRNAs

**Fig. 3:**
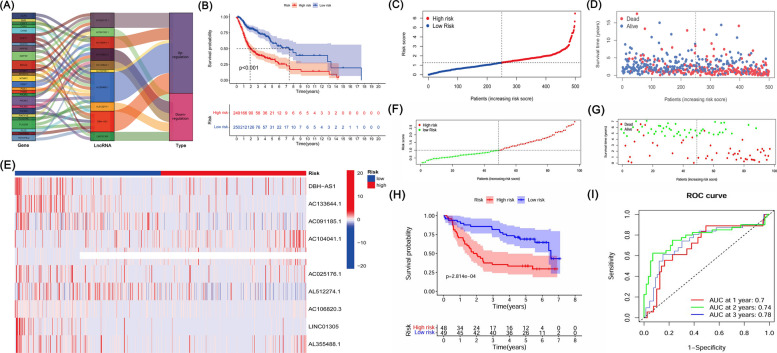
Prognostic significance of the 10-lncRNA model related to phospholipid metabolism. **A** Sankey diagram illustrating the associations between risk lncRNAs and their target genes. **B** Kaplan–Meier survival curves comparing high-risk and low-risk groups in HNSCC patients. **C** Visualization of the phospholipid metabolism-associated lncRNA risk model. **D** Distribution of survival time and survival status among patients. **E** Heatmap showing the expression profiles of the 10 identified risk-associated lncRNAs. **F** Distribution of risk scores among all patients. **G** Distribution of survival status (alive or deceased) among all patients. **H** Kaplan–Meier survival curves comparing overall survival between the high-risk and low-risk groups. **I** Time-dependent receiver operating characteristic (ROC) curves for predicting 1-, 2-, and 3-year overall survival

We identified a total of 1,361 lncRNAs related to phospholipid metabolism. Through univariate Cox analysis, 64 phospholipid metabolism-related lncRNAs were found to have significant prognostic value and were subsequently included in the multivariate Cox analysis. Ultimately, 10 differentially expressed lncRNAs (AC104041.1, AC106820.3, AL355488.1, LINC01305, AC133644.1, AC091185.1, AL158166.1, AL512274.1, DBH-AS1, AC025176.1) were identified as independent prognostic markers for HNSCC (Table 2). The risk score was computed using the corresponding coefficients for these 10 phospholipid metabolism-related lncRNAs according to the following formula: risk score = (0.3288 × AC104041.1 expression) + (−0.6807 × AC106820.3 expression) + (0.5160 × AL355488.1 expression) + (−0.4073 × LINC01305 expression) + (−0.8125 × AC133644.1 expression) + (−0.9886 × AC091185.1 expression) + (0.1550 × AL158166.1 expression) + (−0.0344 × AL512274.1 expression) + (0.3893 × DBH-AS1 expression) + (0.4915 × AC025176.1 expression) (Fig. 3A). Subsequently, patients with HNSCC were categorized into two groups, namely high-risk and low-risk groups, based on the median risk score.

**Table 2:** LncRNA list and coefficient

LncRNA	Coefficient	Hazard ratio (95% CI)	*P* value
AC104041.1	0.328777157	1.39(1.11–1.73)	0.003
AC106820.3	−0.680740091	0.51(0.32–0.81)	0.004
AL355488.1	0.515951787	1.68(1.15–2.44)	0.007
LINC01305	−0.407341407	0.67(0.49–0.90)	0.009
AC133644.1	−0.812511884	0.44(0.23–0.85)	0.014
AC091185.1	−0.988592654	0.37(0.17–0.82)	0.014
AL158166.1	0.155026168	1.17(1.03–1.33)	0.019
AL512274.1	−0.034454485	0.97(0.94–0.99)	0.020
DBH-AS1	0.389278698	1.48(1.04–2.09)	0.027
AC025176.1	0.491475571	1.63(1.01–2.64)	0.044

### Validation of the model

Kaplan–Meier analysis revealed that high-risk lncRNA signatures were significantly linked to poorer survival outcomes (*P* < 0.001, Fig. 3B). Analysis of patient risk scores alongside survival status plots indicated an inverse relationship between risk score and survival in HNSCC patients (Fig. 3C-D). Notably, our heatmap highlighted that certain novel lncRNAs identified in this study exhibited a negative association with our risk model, suggesting the need for further exploration (Fig. 3E). Based on the established prognostic model, risk scores were calculated for all samples in the training cohort, along with their corresponding survival status. The results demonstrated considerable heterogeneity in risk scores among patients, with higher risk scores being associated with an increased number of death events (Fig. 3F). These findings suggested a potential association between the risk score and clinical outcomes in HNSCC. Subsequently, patients in the training cohort were divided into high-risk (above the median value) and low-risk (below the median value) groups according to the median risk score. Kaplan–Meier survival analysis revealed that patients in the low-risk group exhibited significantly better overall survival than those in the high-risk group (*P* < 0.05; Fig. 3G), further supporting the notion that higher risk scores were associated with poorer prognosis in HNSCC. In addition, time-dependent ROC analysis demonstrated that the AUC values for 1-, 2-, and 3-year survival ranged from 0.70 to 0.78, indicating that the constructed model possessed good predictive performance for survival outcomes in HNSCC (Fig. 3H–I).

**Fig. 4:**
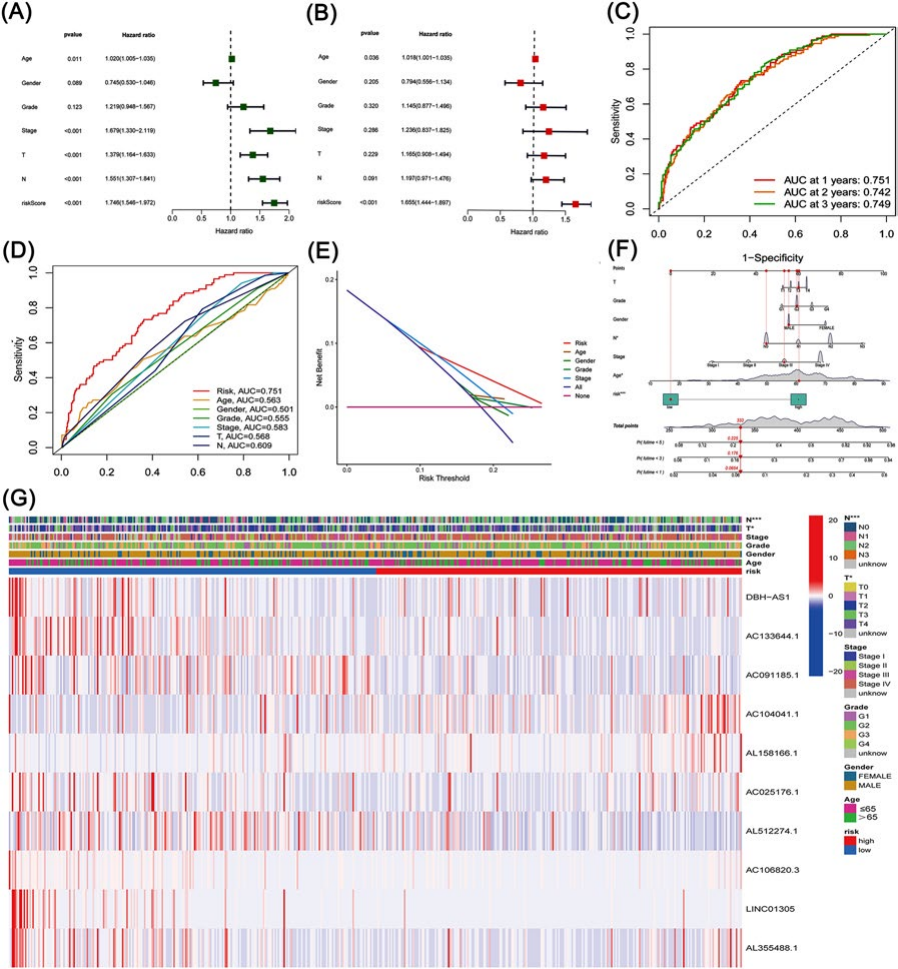
Clinical evaluation of the risk prediction model. **A** Univariate Cox regression analysis assessing clinical variables alongside the risk score. **B** Multivariate Cox regression analysis of clinical variables and the risk score for prognostic evaluation. **C** Area Under the Curve (AUC) values for 1-, 3-, and 5-year ROC curves, illustrating the model's predictive accuracy. **D** Comparison of the 1-year ROC curve of the risk model against other clinicopathological factors. **E** Decision Curve Analysis (DCA) comparing the clinical utility of the risk model with other clinicopathological characteristics. **F** Nomogram incorporating prognostic indicators for predicting 1-, 3-, and 5-year survival probabilities in HNSCC patients. **G** Heatmap showing the correlation between the risk model and various clinicopathological parameters

Subsequently, age, clinical stage, T stage, N stage, and risk score were identified as significant predictors of overall survival (Fig. 4A). Due to incomplete M stage data, it was excluded from the analysis. Furthermore, as depicted in Fig. 4B, multivariate Cox regression confirmed the risk score as an independent and reliable prognostic factor for HNSCC patients. ROC analysis evaluated the model’s sensitivity and specificity in prognosis prediction, with AUC values of 0.751, 0.742, and 0.749 for 1-year, 2-year, and 3-year survival, respectively, indicating robust sensitivity for survival prediction (Fig. 4C). Additionally, the model's AUC of 0.751 reflected its superior prognostic performance compared to conventional clinicopathological features (Fig. 4D-E). To visually estimate individual survival probabilities, we constructed a nomogram incorporating prognostic factors, including gender, age, grade, clinical stage, T stage, N stage, and the risk score, which predicted the 1-, 3-, and 5-year survival rates for HNSCC patients (Fig. 4F). Furthermore, we analyzed a heatmap illustrating associations between lncRNAs, the phospholipid metabolism-related lncRNA prognostic signature, and clinicopathological characteristics. The risk score demonstrated strong correlations with clinical stage, T stage, and N stage (Fig. 4G).

### GSEA

**Fig. 5:**
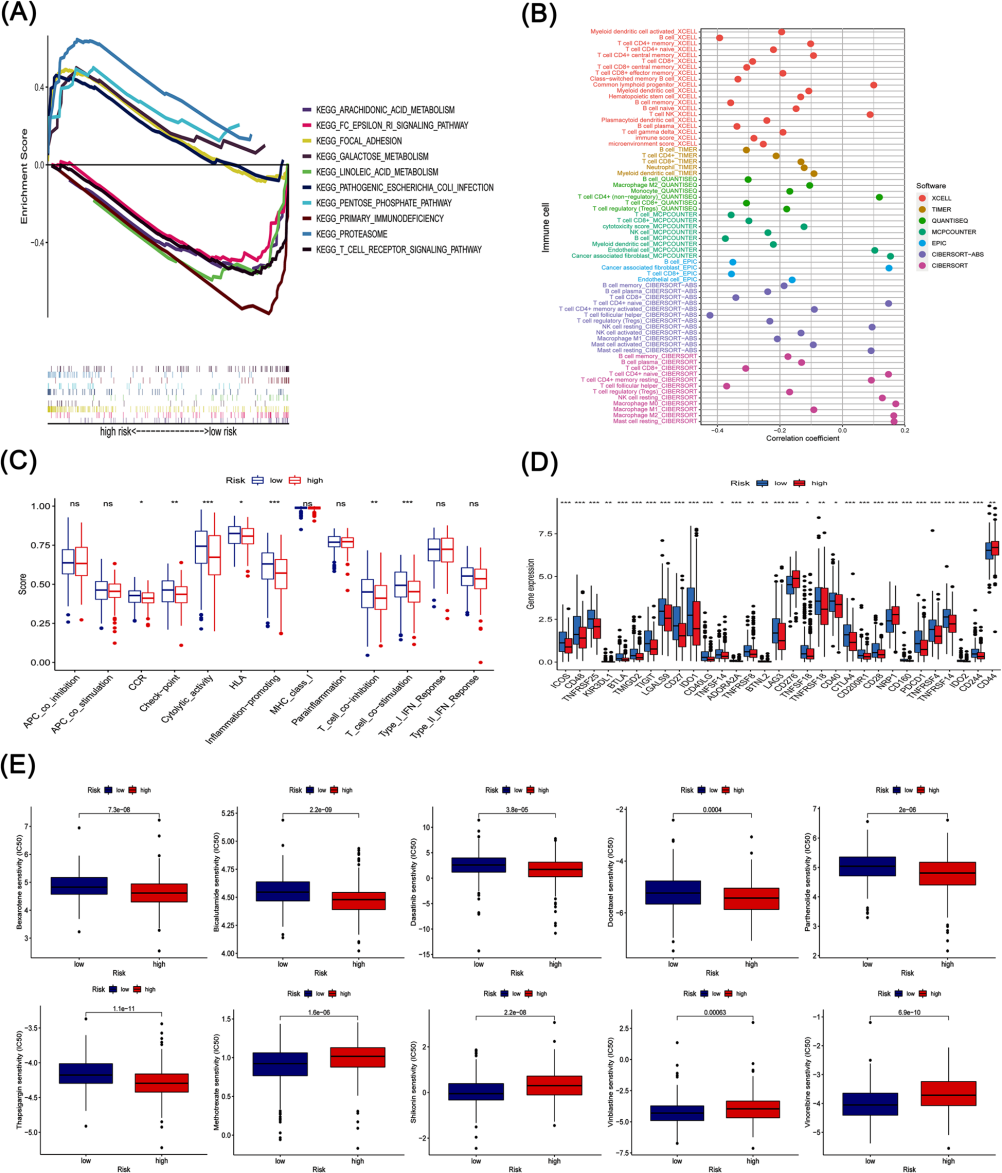
Comprehensive analysis of immune-related gene set enrichment. **A** Results of the GSEA analysis. **B** Correlation analysis between risk scores and immune cell infiltration. **C** Comparative analysis of immune function scores between high-risk and low-risk groups. **D** Differential expression of immune checkpoint molecules between high-risk and low-risk groups. **E** Drug sensitivity comparison between high-risk and low-risk groups

To gain deeper insights into the molecular mechanisms linked to the prognostic signature of phospholipid metabolism-related lncRNAs, we performed Gene Set Enrichment Analysis (GSEA). The GSEA findings indicated that pathways such as proteasome, focal adhesion, pathogenic Escherichia coli infection, galactose metabolism, and the pentose phosphate pathway were predominantly enriched in the high-risk group. In contrast, pathways including arachidonic acid metabolism, primary immunodeficiency, T cell receptor signaling pathway, linoleic acid metabolism, Fc epsilon RI signaling pathway, aldosterone-regulated sodium reabsorption, alpha-linolenic acid metabolism, B cell receptor signaling pathway, intestinal immune network for IgA production, fatty acid metabolism, Fc gamma R-mediated phagocytosis, JAK-STAT signaling pathway, VEGF signaling pathway, retinol metabolism, amyotrophic lateral sclerosis (ALS), pantothenate and CoA biosynthesis, and ether lipid metabolism were mainly enriched in the low-risk group (Fig. 5A).

### Immunity analysis of the model

We performed a detailed analysis of the relationship between the risk score and immune infiltration. B cells, CD8 + T cells, CD4 + T cells, and NK cells demonstrated negative correlations with the risk score, whereas common lymphoid progenitors, NK T cells, endothelial cells, resting mast cells, and M0 macrophages showed positive correlations with the risk score (Fig. 5B). Further, we applied single-sample Gene Set Enrichment Analysis (ssGSEA) to examine differences in immune functions between the two groups. Patients in the low-risk group exhibited enhanced immune-related activities, particularly in areas such as CCR, checkpoint signaling, cytolytic activity, HLA expression, inflammation promotion, T cell co-inhibition, and T cell co-stimulation (Fig. 5C). Given the relevance of immune checkpoint inhibitor therapies, we also assessed the association between the risk score and the expression of key immune checkpoint molecules. This analysis revealed significant differences in the expression of ICOS, CD48, IDO1, CD276, CD40, NRP1, and CD44 between the two patient groups. Additionally, CD276, NRP1, and CD44 were more highly expressed in the high-risk group, suggesting an immunosuppressed and exhausted phenotype in this cohort of patients (Fig. 5D).

### Drug sensitivity analysis

To enhance the effectiveness of therapeutic interventions for HNSCC patients, we conducted a detailed investigation into the differences in sensitivity to common chemotherapy drugs between the two patient groups. The results of our drug sensitivity analysis revealed that the IC50 values of drugs, including bexarotene, bicalutamide, dasatinib, docetaxel, parthenolide, and thapsigargin, were lower in the high-risk group compared to the low-risk group. Conversely, the IC50 values of methotrexate, shikonin, vinblastine, and vinorelbine were significantly lower in the low-risk group compared to the high-risk group, indicating that patients in the low-risk group exhibited increased sensitivity to these drugs (Fig. 5E).

### Cluster analysis based on prognostic phospholipid metabolism-related lncRNAs

**Fig. 6:**
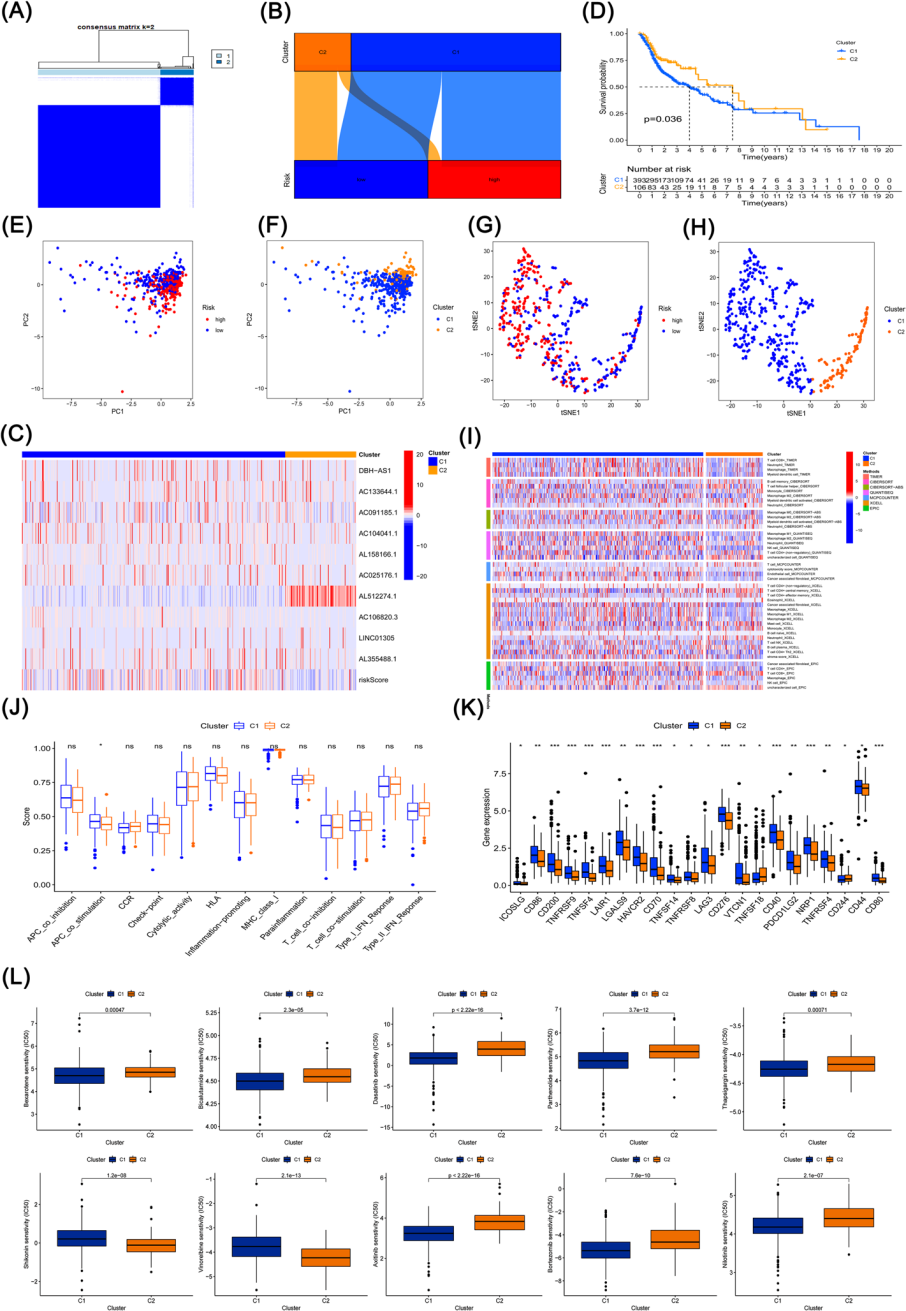
Clustering analysis of patient subgroups. **A** Patient classification into two clusters using the ConsensusClusterPlus algorithm. **B** Sankey diagram illustrating the relationship between clusters and risk groups. **C** Heatmap showing expression profiles of the 10 prognostic phospholipid metabolism-related lncRNAs. **D** Kaplan–Meier survival analysis comparing overall survival between clusters 1 and 2 in HNSCC patients. **E** Principal component analysis (PCA) of the defined risk groups. (F) PCA of the identified clusters. **G** t-SNE plot visualizing the distribution of risk groups. **H** t-SNE plot illustrating the separation of clusters. **I** Heatmap depicting immune cell distribution across clusters. **J** Comparative immune function scores between clusters 1 and 2. **K** Expression levels of immune checkpoint markers between the two clusters. (L) Drug sensitivity analysis comparing clusters 1 and 2

We conducted an in-depth analysis of HNSCC patient clusters to compare immune microenvironments and responses across distinct tumor subtypes. Utilizing the ten phospholipid metabolism-related lncRNAs comprising the risk model, we applied the ConsensusClusterPlus R package to classify patients into two clusters (Fig. 6A). As shown in Fig. 6B-C, most patients in the high-risk group were allocated to cluster 1, whereas cluster 2 predominantly comprised patients from the low-risk group. Notably, survival analysis indicated that cluster 2 had a significantly better overall survival (OS) than cluster 1 (Fig. 6D). Our PCA findings (Fig. 6E-F) demonstrated that the risk groups and clusters formed distinct principal components, further corroborated by t-SNE analysis, which validated the separation between the two clusters (Fig. 6G-H). Immune infiltration analysis revealed differences in immune cell infiltration patterns across clusters, as displayed in the heatmap (Fig. 6I). Immune function analysis highlighted a significant difference in APC co-stimulation between clusters 1 and 2 (Fig. 6J). In terms of immune checkpoint analysis, genes such as TNFRSF9, LAG3, CD276, CD40, NRP1, and CD44 showed higher expression levels in cluster 1 (Fig. 6K). Furthermore, drug sensitivity analysis indicated that cluster 1 exhibited increased sensitivity to agents like bexarotene, bicalutamide, dasatinib, parthenolide, and thapsigargin. Conversely, cluster 2 displayed lower IC50 values for shikonin and vinorelbine. There were no significant differences in IC50 values for docetaxel, methotrexate, and vinblastine between the clusters. Additionally, differential drug responses for agents like axitinib, bortezomib, and nilotinib were observed between clusters 1 and 2, with no notable differences detected between high- and low-risk groups (Fig. 6L).

### Knockdown of AL158166.1 inhibited proliferation, migration, invasion and phospholipid metabolism of HNSCC cells

**Fig. 7:**
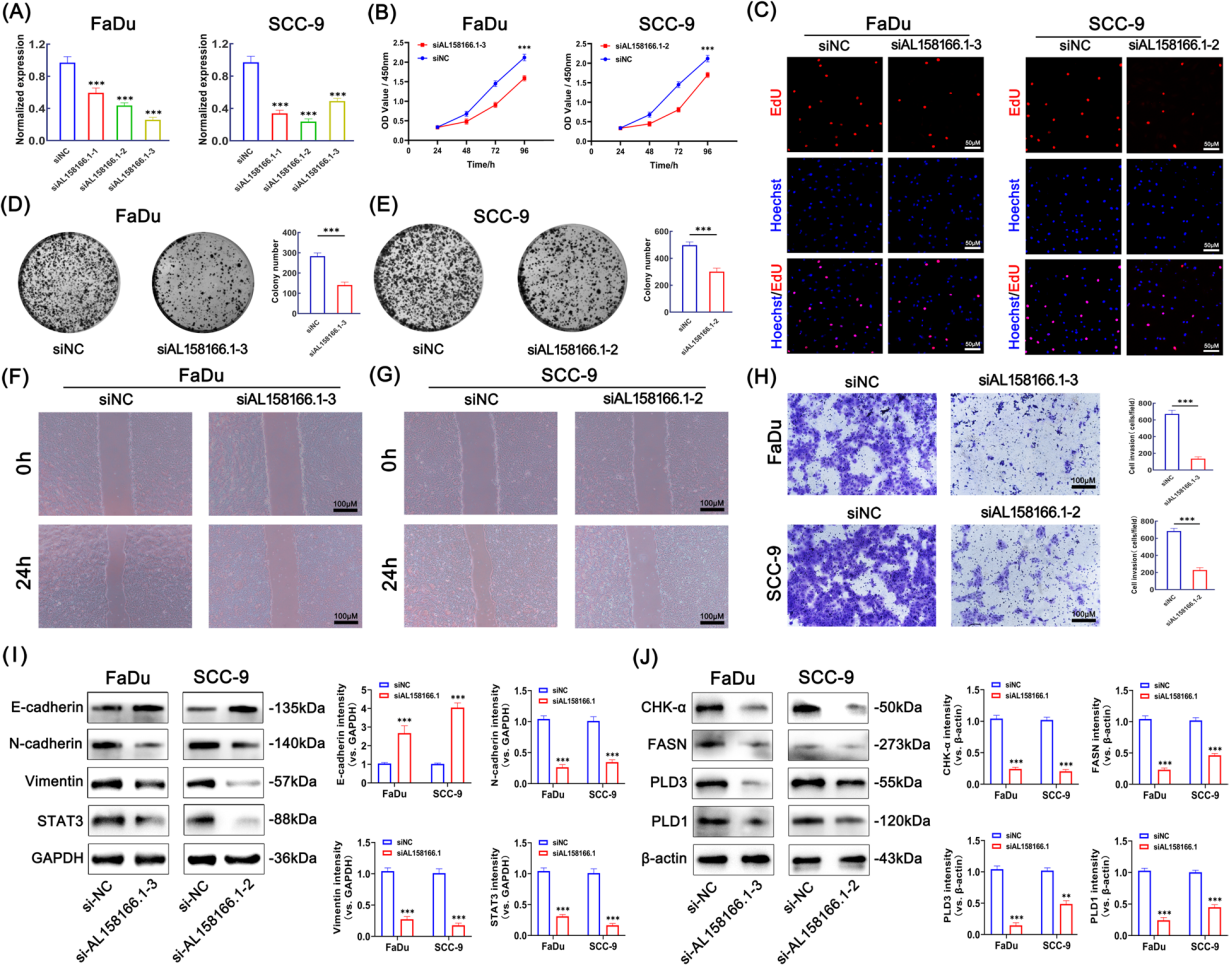
Inhibition of AL158166.1 inhibited proliferation, migration, invasion and phospholipid metabolism of HNSCC cells. **A** Quantification of AL158166.1 expression levels in FaDu and SCC-9 cells transfected with either negative control or siRNA was performed using qRT-PCR. Expression levels were normalized to GAPDH, and results were presented as fold changes relative to the negative control. **B** The CCK-8 assay was used to evaluate the effect of AL158166.1 knockdown on the proliferation of FaDu and SCC-9 cells (*n* = 5). **C** The EdU staining assay and subsequent quantification were conducted to evaluate the effect of AL158166.1 knockdown on the proliferation of FaDu and SCC-9 cells. The scale bar is set at 50 μm (*n* = 3). **D**-**E** The colony formation assay was conducted to further investigate the effect of AL158166.1 knockdown on the proliferation of FaDu and SCC-9 cells (n = 3). **F**-**G** Wound-Healing test was used to determine the role of AL158166.1 siRNA in FaDu and SCC-9 cell migration. The scale bar is set at 100 μm (n = 3). **H** Transwell assays and subsequent quantification were used to determine the role of AL158166.1 siRNA in FaDu and SCC-9 cell invasion. The scale bar is set at 100 μm (*n* = 3). **I** The influence of AL158166.1 on the protein expression levels of E-cadherin, N-cadherin, vimentin, and STAT3 in FaDu and SCC-9 cells was evaluated using western blotting and subsequent quantification (*n* = 3). **J** The knockdown of AL158166.1 on the protein expression levels of CHK-α, FASN, PLD3, and PLD1 in FaDu and SCC-9 cells was evaluated using western blotting and subsequent quantification (*n* = 3). All data represent mean ± SD of three independent experiments; *p < 0.05, **p < 0.01, ***p < 0.001

Our heatmap analysis revealed that AL158166.1 was markedly upregulated in the high-risk group (Fig. 3E). Among the ten phospholipid metabolism-related lncRNAs included in the prognostic signature, AL158166.1 was therefore prioritized for functional validation due to its prominent contribution to the risk model, its robust association with patient prognosis and risk stratification, as well as its consistent relevance to immune-related and therapeutic response analyses, together with favorable experimental feasibility and biological interpretability. To further investigate the impact of AL158166.1 on HNSCC tumorigenesis, we conducted multiple experiments to validate the effect of AL158166.1 knockdown on the malignant characteristics of HNSCC cells. Following the confirmation of the efficacy of the three AL158166.1 siRNAs in FaDu and SCC-9 cells through quantitative RT-PCR, siAL158166.1–3 and siAL158166.1–2 were chosen for subsequent functional experiments (Fig. 7A). To assess the influence of AL158166.1 on the malignant characteristics of HNSCC cells, we examined the proliferation and clonogenic ability of FaDu and SCC-9 cells following AL158166.1 knockdown using CCK-8, EdU staining, and colony formation assays. As shown in Fig. 7B-E, knockdown of AL158166.1 significantly reduced the proliferation and colony formation of FaDu and SCC-9 cells compared to the negative control. Subsequently, we investigated the effect of AL158166.1 on the migration and invasion of HNSCC cells using wound healing and Transwell assays. The results (Fig. 7F-H) indicated that knockdown of AL158166.1 significantly impaired the migration and invasion capabilities of FaDu and SCC-9 cells compared to the negative control. Western blot analysis was performed to evaluate the expression of markers associated with tumor metastasis and invasion, including E-cadherin, N-cadherin, vimentin, and STAT3. As shown in Fig. 7I, AL158166.1 knockdown increased E-cadherin expression, while the expression levels of N-cadherin, vimentin, and STAT3 were significantly reduced. Additionally, we evaluated the expression of phospholipid metabolism-related markers, including CHK-α, FASN, PLD3, and PLD1, using Western blot analysis. As illustrated in Fig. 7J, AL158166.1 knockdown significantly reduced the expression levels of CHK-α, FASN, PLD3, and PLD1 compared to the siNC group. In conclusion, these findings suggest that suppressing AL158166.1 inhibits the proliferation, migration, invasion and phospholipid metabolism of HNSCC cells.

### Knockdown of AL158166.1 inhibited PI3K/AKT/mTOR signaling pathway

**Fig. 8:**
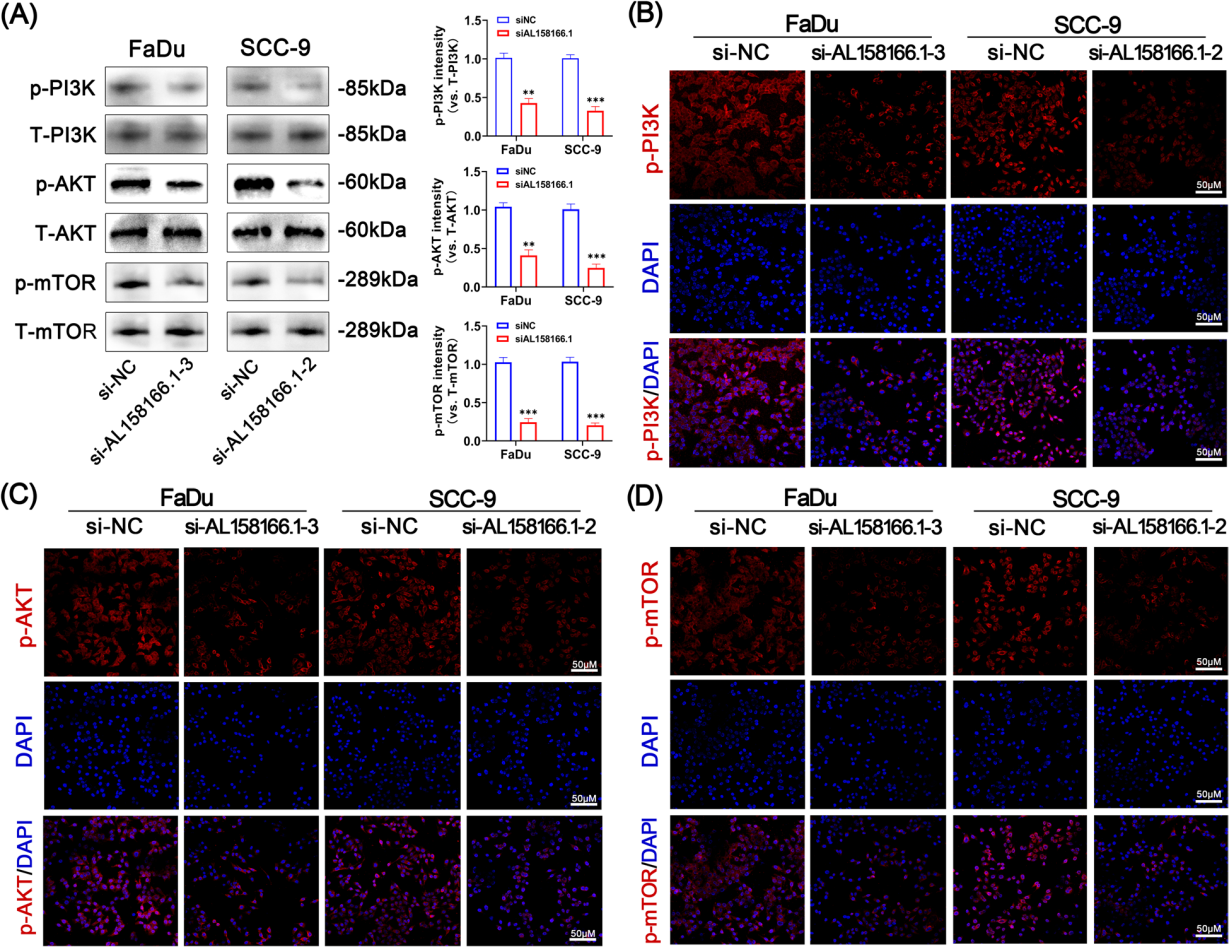
Suppression of the PI3K/AKT/mTOR Signaling Pathway by AL158166.1 Knockdown. **A** Western blot analysis and subsequent quantification were employed to evaluate the impact of AL158166.1 on the expression levels of p-PI3K, T-PI3K, p-AKT, T-AKT, p-mTOR, and T-mTOR in FaDu and SCC-9 cells. **B** The effect of AL158166.1 on the protein expression levels of p-PI3K in FaDu and SCC-9 cells was evaluated using immunofluorescence staining. Magnification × 400. The scale bar is set at 50 μm (*n* = 3). **C** p-AKT expression in FaDu and SCC-9 cells was determined using immunofluorescence staining. Magnification × 400. The scale bar is set at 50 μm (*n* = 3). **D** The effect of ajugol on the protein expression levels of p-mTOR in FaDu and SCC-9 cells was assessed through immunofluorescence staining (scale bar, 50 μm; *n* = 3). All data are presented as mean ± standard deviation (SD) from three independent experiments; *p < 0.05, **p < 0.01, ***p < 0.001

GSEA analysis showed that focal adhesion pathway was mainly enriched in the high-risk group. Research has demonstrated that signaling molecules PI3K and AKT can facilitate cancer cell adhesion and migration [30]. Scientific literature documents that the PI3K/AKT/mTOR signaling pathway governs a wide array of vital cellular processes crucial for maintaining health, including metabolism, cell proliferation, apoptosis, angiogenesis and the cell cycle, etc. In order to further confirm whether the mechanism of AL158166.1 promoting the malignant phenotype of HNSCC cells is related to the PI3K/AKT/mTOR signaling pathway, we investigated the involvement of this pathway using Western blot analysis. As shown in Fig. 8A, in comparison to the control group, knockdown of AL158166.1 inhibits the activation of the PI3K/AKT/mTOR pathway. The expression levels of p-PI3K, p-AKT, and p-mTOR in FaDu and SCC-9 cells from each group were subsequently evaluated using immunofluorescence staining. As shown in Fig. 8B-D, compared with the si-NC group, knockdown of AL158166.1 significantly inhibited the activation of PI3K, AKT, and mTOR. Therefore, we hypothesized that AL158166.1 promotes the malignant phenotype of HNSCC cells by activating the PI3K/AKT/mTOR pathway.

## Discussion

HNSCC is a prevalent malignancy that may arise in various regions within the head and neck area [31]. Despite significant advancements in cancer therapies, the general prognosis for HNSCC remains disheartening due to the absence of reliable predictive markers [7]. Phospholipid metabolism provides essential energy and structural components critical for cellular life processes [32]. Phospholipid metabolites play an essential role in defining cellular structures and orchestrating signaling pathways that govern various malignant characteristics, including proliferation, invasion, migration, stemness, and drug resistance [10]. Consequently, incorporating phospholipid metabolism-related biomarkers into a unified prognostic model, while evaluating its predictive accuracy, immune implications, and responsiveness to targeted therapies, holds significant potential to individualize treatment plans and improve therapeutic efficacy. Over the past decade, lncRNAs have been the subject of extensive investigation [33]. This is primarily due to its critical role in tumor growth, metastasis, and chemotherapy resistance [28]. Numerous studies have investigated the associations between lncRNAs and HNSCC prognosis, thereby advancing the understanding of clinical outcomes. Thus, examining the expression of phospholipid metabolism-related lncRNAs in HNSCC for novel prognostic biomarkers and therapeutic targets is highly significant.

In this study, we employed bioinformatics techniques to conduct a comprehensive examination of lncRNAs associated with phospholipid metabolism in HNSCC. Our analysis identified 31 DEGs related to phospholipid metabolism. GO analysis indicated that these genes were significantly involved in the glycerophospholipid metabolic process, predominantly located within the phosphatidylinositol 3-kinase complex, and primarily enriched in phosphatidylinositol phosphate kinase activity. Glycerophospholipids (GPLs) have been shown to regulate various cellular functions, including adhesion, migration, apoptosis, and signal transduction [34]. The phosphatidylinositol-3-kinase (PI3K) pathway is widely recognized as one of the most commonly activated signaling pathways in human cancers. It functions as a critical downstream component of receptor tyrosine kinases (RTKs) and plays a pivotal role in regulating various cellular processes, including proliferation, growth, differentiation, migration, and survival [35]. The KEGG pathway enrichment analysis similarly indicated that the differentially expressed genes were chiefly associated with pathways involving glycerophospholipid metabolism and phosphatidylinositol signaling.

The findings from our study underscore the importance of 64 lncRNAs associated with phospholipid metabolism in influencing the survival outcomes of HNSCC patients. From this group, we identified a subset of 10 lncRNAs (AC104041.1, AC106820.3, AL355488.1, LINC01305, AC133644.1, AC091185.1, AL158166.1, AL512274.1, DBH-AS1, AC025176.1) to construct a prognostic signature. Of these, five lncRNAs function as oncogenes, while the remaining five exhibit tumor-suppressive properties. For instance, AC104041.1, which is highly expressed in HNSCC patients, acts as a potential oncogenic lncRNA by promoting tumor growth and metastasis. Its oncogenic effects are associated with its interaction with miR-6817-3p, through which AC104041.1 plays a crucial role in enhancing HNSCC cell proliferation and migration [36]. In a similar vein, Zhu et al. explored a risk model featuring AL355488.1 for predicting ovarian cancer prognosis [37]. AL355488.1 has been shown to affect cell proliferation, migration, and invasion in hepatocellular carcinoma through modulation of the hsa-miR-29c-3p-P3H1 axis [38]. LINC01305, another lncRNA identified in our signature, has been associated with promoting cell proliferation and metastasis in esophageal squamous carcinoma by interacting with IGF2BP2 and IGF2BP3, which enhances the mRNA stability of HTR3A [39]. Furthermore, LINC01305 has exhibited high expression in cervical cancer (CC) and contributes to tumorigenesis through the miR-129-5p/Sox4 axis [40]. It contributes to CC progression through interactions with KHSRP and is released via exosomes, influencing the stemness of recipient cells. AC091185.1 is also included in a novel risk model for predicting lung adenocarcinoma prognosis [41, 42]. Similarly, the expression of AL512274.1 has been validated as upregulated in HNSCC tissues using quantitative real-time PCR [43]. LncRNA DBH-AS1 has proven to play a pivotal role in the biological progression of various tumors, promoting hepatocellular carcinoma development via the miR-138/FAK/Src/ERK pathway [44]. Moreover, this lncRNA has been recognized as a prognostic biomarker in patients with colorectal cancer, cervical cancer, and melanoma. Previous studies have demonstrated that AC025176.1 can influence the prognosis of hepatocellular carcinoma by regulating N6-methyladenosine (m6A) modification [45]. However, in our study, LINC01305, AC091185.1, and AL512274.1 appear to function as protective factors, which may be attributed to tumor heterogeneity or other contributing factors. Additionally, Qian et al. reported that AL158166.1 acts as a protective factor in laryngeal squamous cell carcinoma [46]. However, in Zhu et al.'s study, this lncRNA appears to function as a cancer-promoting factor in hepatocellular carcinoma [47]. Our experimental findings indicate that the suppression of AL158166.1 significantly inhibits the proliferation, migration, and invasion of HNSCC cells. Moreover, AL158166.1 appears to play a pivotal role in promoting the adhesion and migration of HNSCC cells, primarily through the activation of the PI3K/AKT/mTOR pathway. However, further in-depth research is needed to fully unravel its functional mechanisms. In contrast, several lncRNAs in our study, such as AC106820.3 and AC133644.1, have limited documentation regarding their functions. Therefore, more extensive investigations are required to elucidate the roles and mechanisms of these lncRNAs. Additionally, the Sankey diagram in our study underscores the significance of several lncRNA-associated genes in various tumor biological processes. For example, PIK3R3, a regulatory subunit of PI3K, is known to promote metastasis in pancreatic cancer by facilitating ZEB1-induced epithelial-mesenchymal transition [48]. These findings underscore the complex and multifaceted nature of lncRNA-related mechanisms in tumor progression, highlighting the need for further exploration.

Utilizing the expression levels of the 10 identified phospholipid metabolism-related lncRNAs, we computed a risk score for each tumor sample, thereby establishing a prognostic signature. Our analysis of the relationship between the risk score, survival status, and overall survival outcomes demonstrated the effectiveness of our prognostic model in accurately predicting the prognosis of HNSCC patients. Univariate and multivariate Cox regression analyses further confirmed that our signature could serve as an independent prognostic marker, regardless of variables such as gender, age, tumor grade, clinical stage, T stage, and N stage. This finding highlights the intrinsic prognostic significance of phospholipid metabolism-related lncRNAs. Moreover, the development of a novel nomogram holds great potential for enhancing clinical decision-making and providing valuable guidance for individualized treatment strategies. In addition, the results of the GSEA analysis revealed that our signature was primarily associated with pathways related to metabolism and immunity. These pathways included galactose metabolism, arachidonic acid metabolism, primary immunodeficiency, and the T cell receptor signaling pathway. Consequently, our signature not only demonstrates the ability to predict the prognosis of HNSCC patients but also potentially holds significant relevance in the biological mechanisms underlying HNSCC.

The modulation of the immune system plays a crucial role in influencing the development of HNSCC. Factors such as the types and ratios of immune-infiltrating cells, the manifestation of immune functions, and the activation of immune checkpoints are all critical in determining the response to immunotherapy and the prognosis of patients. Our analysis revealed a strong negative correlation between the risk score and the presence of immune cells, including CD8 + T cells, CD4 + T cells, NK cells, and B cells, indicating a higher abundance of these immune cells in low-risk samples. CD8 + T cells, as the primary effector cells involved in anti-tumor immune responses, are associated with improved patient survival [49]. Similarly, CD4 + T cells play a crucial role in supporting CD8 + cytotoxic T lymphocytes to target and eliminate tumor cells [50]. NK cells and B cells also play a significant role in tumor control [51, 52]. Therefore, variations in the composition of these immune-infiltrating cells likely contribute to the observed disparities in survival between the high-risk and low-risk groups.

Furthermore, we observed elevated expression levels of immune checkpoint genes, including CD276, NRP1, and CD44, in the high-risk group. CD276 has been associated with facilitating the evasion of immune surveillance by cancer stem cells (CSCs) throughout the initiation, progression, and metastasis of HNSCC. The use of monoclonal antibodies to target CD276 has demonstrated efficacy in eradicating CSCs and restraining tumor growth and metastasis, primarily by augmenting CD8 + T lymphocyte-mediated anti-tumor immunity [53]. NRP1 limits the reinvigoration of CD8 + T cells in the presence of checkpoint inhibitors, posing a barrier to the sustained effectiveness of CD8 + T cell-mediated tumor immune surveillance [54]. Elevated levels of CD44 have been observed in HNSCC cell lines and are associated with enhanced cell migration [55]. It is essential to further investigate the functional roles of these immune checkpoints in cancer and to explore the potential therapeutic benefits of targeting these checkpoints or their signaling pathways.

Moreover, tumor samples underwent consensus clustering analysis based on lncRNA expression levels, resulting in their division into two clusters, cluster 1 and cluster 2. Notably, cluster 1 exhibited a higher risk score and worse survival outcomes compared to cluster 2, aligning with the observation that cluster 1 was enriched with a greater proportion of high-risk samples. Similarly, stratifying the HNSCC dataset into these two subtypes revealed that cluster 2, primarily composed of low-risk patients, showed a higher infiltration of immune cells, suggesting that individuals in this cluster may have a more favorable response to cancer immunotherapies. In addition, we conducted a drug sensitivity analysis focusing on IC50 values. The results indicated that high-risk patients demonstrated increased sensitivity to bexarotene, bicalutamide, dasatinib, docetaxel, parthenolide, and thapsigargin. Conversely, cluster 2 showed greater sensitivity to shikonin and vinorelbine, while cluster 1 exhibited enhanced sensitivity to bexarotene, bicalutamide, dasatinib, parthenolide, and thapsigargin. Notably, several of these agents are closely related to existing or exploratory therapeutic strategies in HNSCC. For example, docetaxel and vinorelbine are commonly used or investigated chemotherapeutic agents in HNSCC, particularly in advanced or recurrent settings, while dasatinib and bicalutamide have been explored in preclinical or clinical studies targeting aberrant signaling pathways in head and neck cancers. Although some compounds remain investigational, these findings suggest that both the risk model and tumor subtypes may help inform personalized chemotherapy strategies and guide future translational research in HNSCC. From a clinical perspective, this prognostic model may offer practical guidance for personalized treatment selection in HNSCC. Specifically, high-risk patients, characterized by poor prognosis, reduced immune infiltration, and elevated immune checkpoint expression, may benefit from intensified therapeutic strategies, including combination chemotherapy or targeted agents, rather than monotherapy. In contrast, low-risk patients with higher immune cell infiltration may be more suitable candidates for immunotherapy-based approaches or treatment de-escalation strategies to reduce toxicity while maintaining efficacy. Therefore, risk stratification based on phospholipid metabolism-related lncRNAs may assist clinicians in tailoring treatment intensity and therapeutic modalities according to individual tumor biology.

Our study has several limitations that warrant consideration. Although the risk signature shows potential as a prognostic indicator, the lack of lncRNA data and clinical information from external cohorts limits the generalizability of our findings. Furthermore, the observed associations between the risk signature and its implications for immunotherapy and chemotherapy require validation through extensive clinical trials with larger sample sizes. Another limitation of this study is that the functional role of AL158166.1 was evaluated exclusively through in vitro experiments. The lack of in vivo validation restricts assessment of its effects on tumor growth and progression within a physiological context, which will be addressed in future studies using appropriate HNSCC animal models. In addition, the current study lacks validation of the 10-lncRNA signature using clinical HNSCC tissue specimens. Verification of the correlation between lncRNA expression levels and patient prognosis at the tissue level would further enhance the robustness and clinical relevance of the model. Future studies will focus on incorporating independent clinical cohorts to validate the prognostic value of these lncRNAs. Nevertheless, the correlation analysis of immune cell dynamics across various platforms may serve as a preliminary form of external validation. Despite these limitations, the phospholipid metabolism-related lncRNA risk model remains a promising and reliable tool. Future studies with larger sample sizes should prioritize exploring the application of immunotherapy and chemotherapy, supported by comprehensive bioinformatics analyses and overall survival data.

## Conclusion

In summary, we identified 10 phospholipid metabolism-related lncRNAs with promising prognostic potential in the context of HNSCC. The establishment of this prognostic model represents a valuable advancement, offering insights into the molecular mechanisms underlying HNSCC tumorigenesis. Furthermore, this model may improve our ability to predict therapeutic outcomes for HNSCC patients, paving the way for the development of tailored and precise treatment strategies for individuals affected by this condition.

## Supplementary Information

Supplementary Material 1.

Supplementary Material 2.

## Data Availability

No datasets were generated or analysed during the current study.
