# Supplementary material for: Development and validation of a phospholipid metabolism-associated lncRNA model for prognostic stratification and therapeutic guidance in HNSCC
Source: World J Surg Oncol. 2026 Mar 12;24:183. doi: 10.1186/s12957-026-04299-2 (PMC13093962; doi:10.1186/s12957-026-04299-2)

Declaration of Western Blot Original Data

To comply with the journal's policy requiring unprocessed Western blot (WB) data, we provide the original WB membranes used in our study. For experimental convenience during antibody incubation and optimal detection of both target proteins and internal controls, we performed a single cut on the membranes after protein transfer but before antibody incubation and signal detection. This allowed simultaneous probing of the target protein and loading control on separate membrane sections.

Importantly, the provided WB images can be assembled into continuous membranes, demonstrating that all sections originate from the same experimental run without data manipulation. In some experiments, membranes were stripped of antibodies and reprobed as specified in the Methods section.

We confirm that these procedures were conducted following standard laboratory protocols to ensure data accuracy, reproducibility, and transparency. Please let us know if further clarification is required.

Original Images– Related to Figure 7I

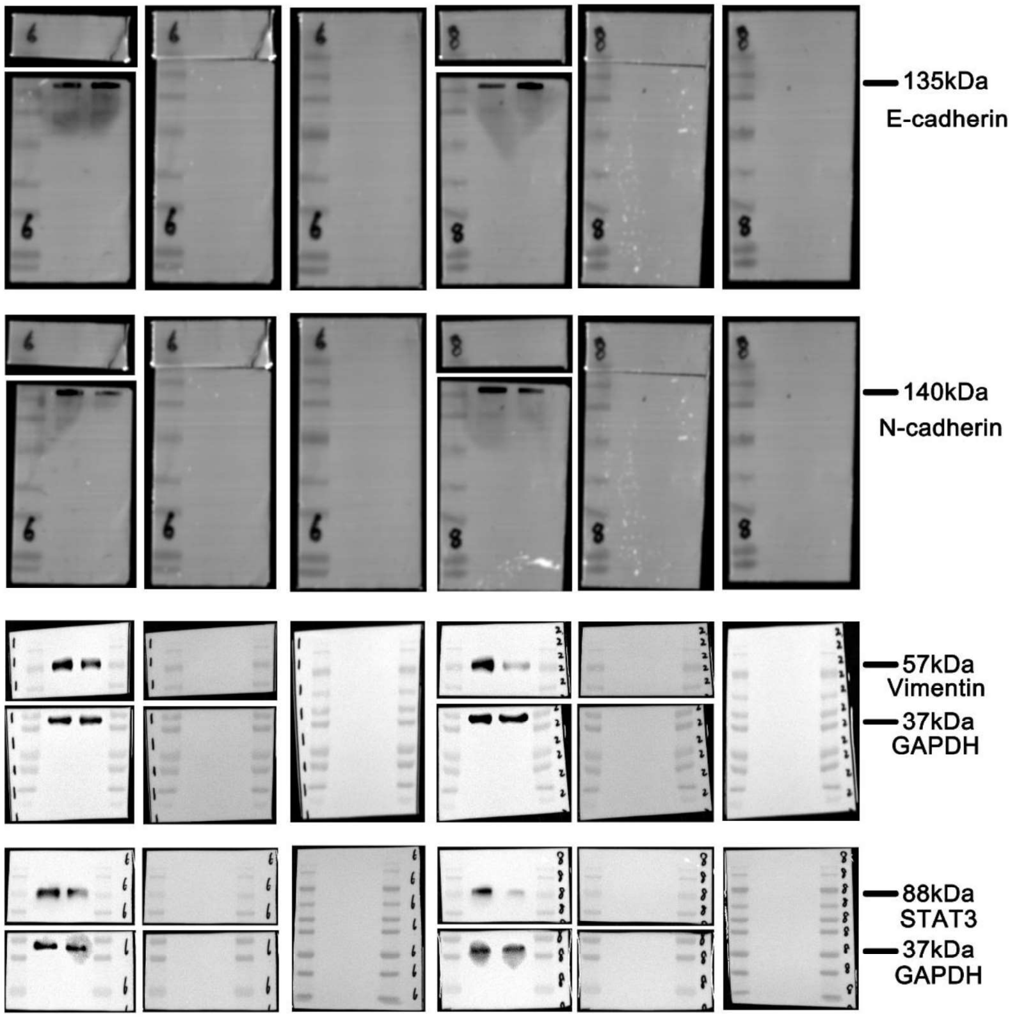

Original Images– Related to Figure 7J

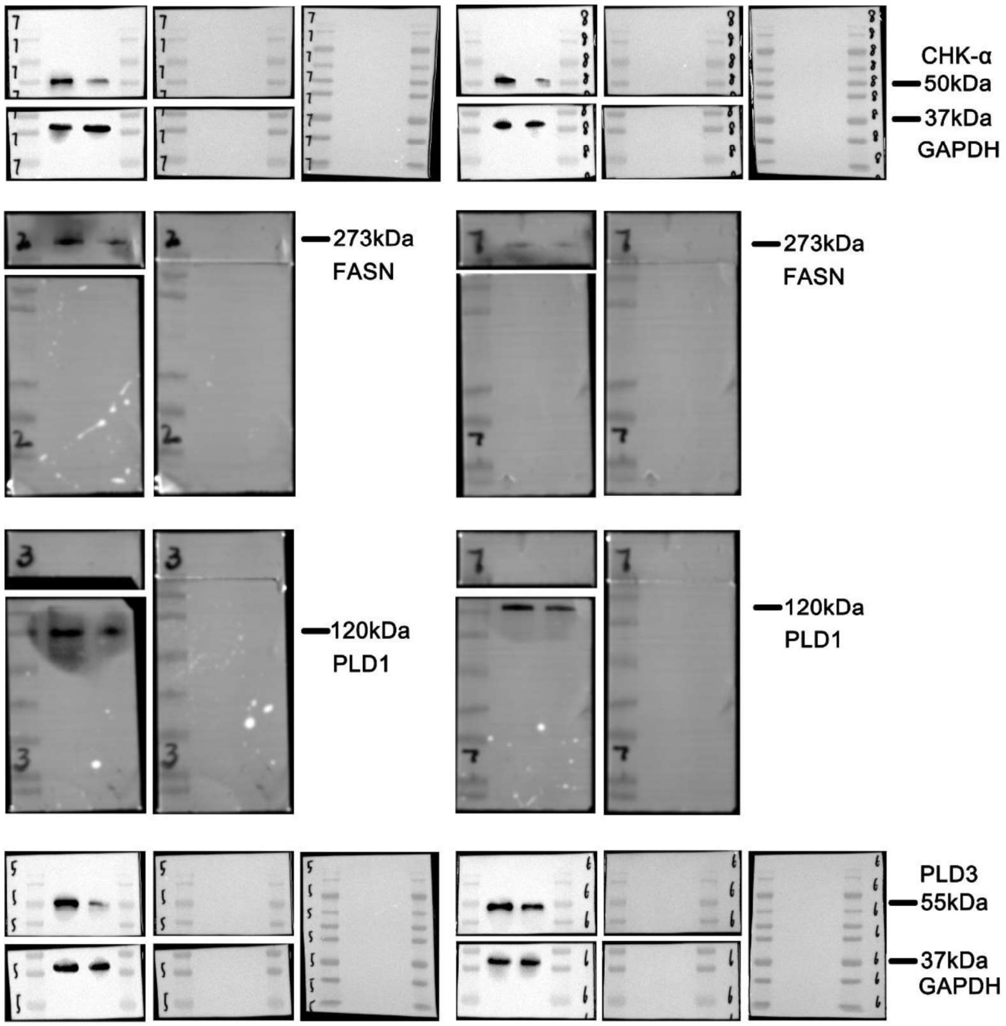

Original Images– Related to Figure 7J

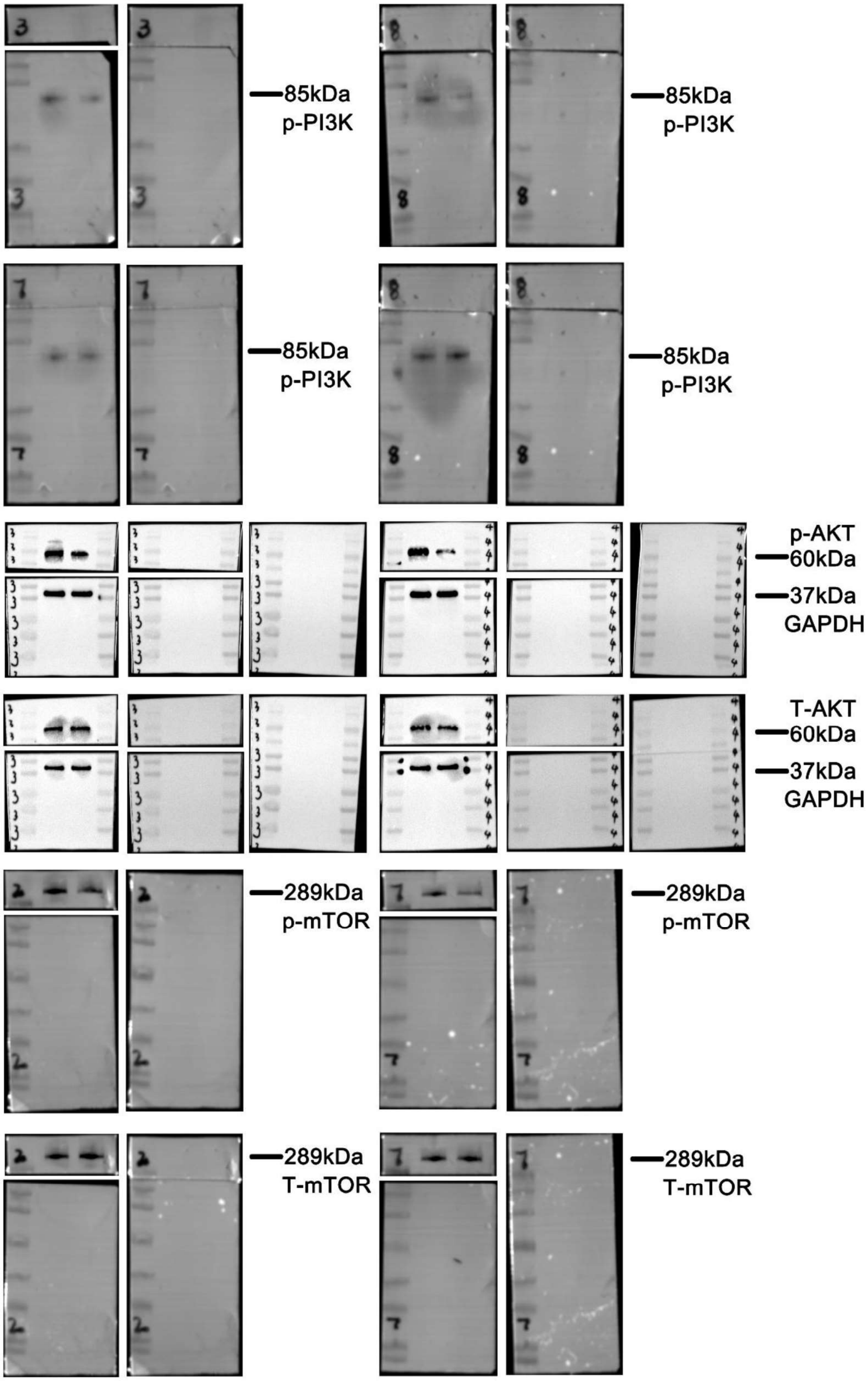

Original Images– Related to Figure 7C

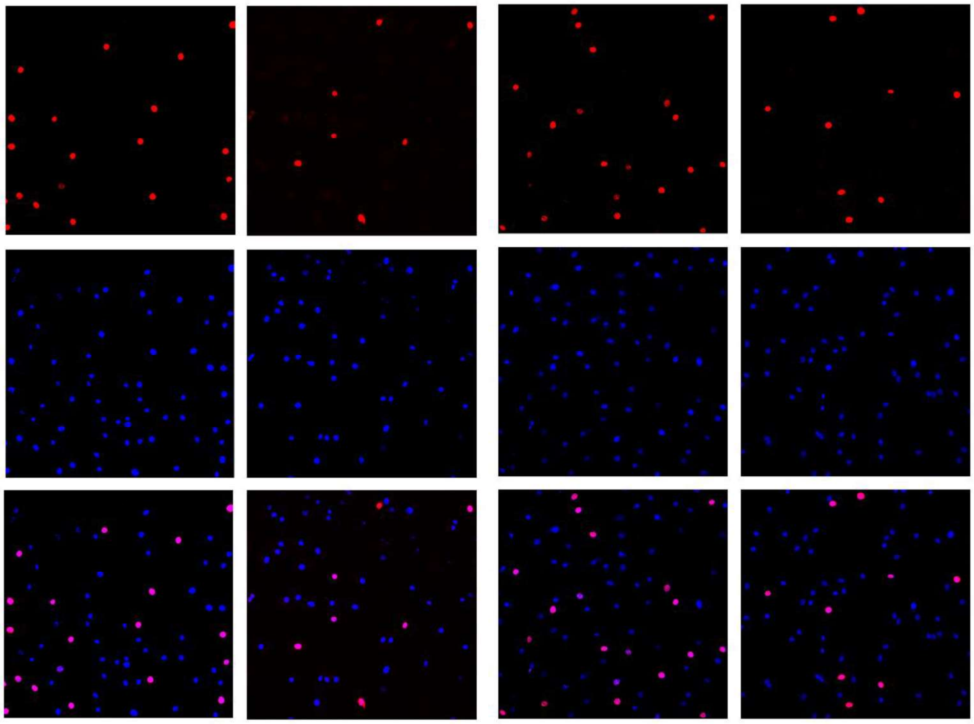

Original Images– Related to Figure 7D-E

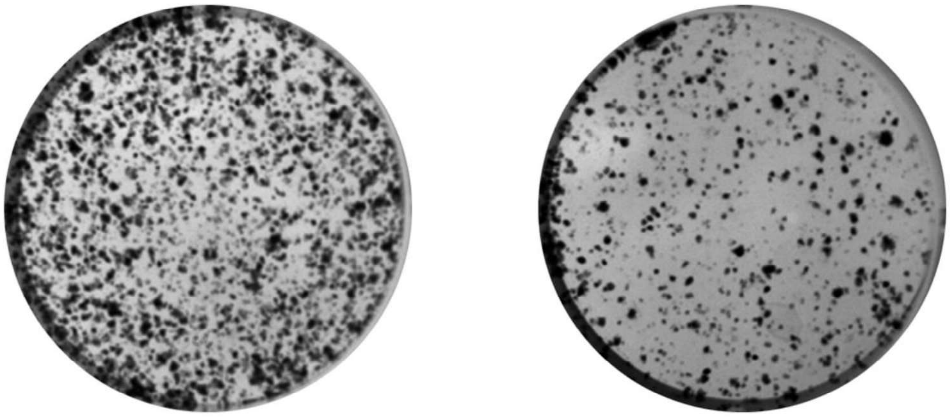

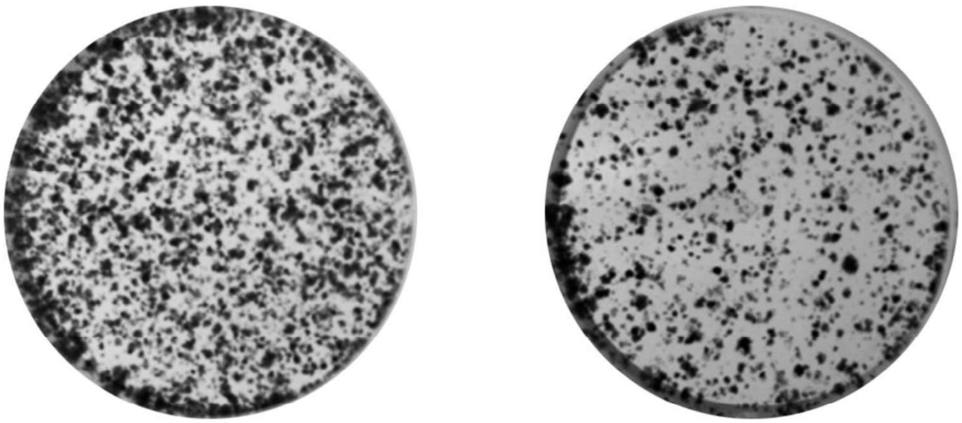

Related to Figure 7F

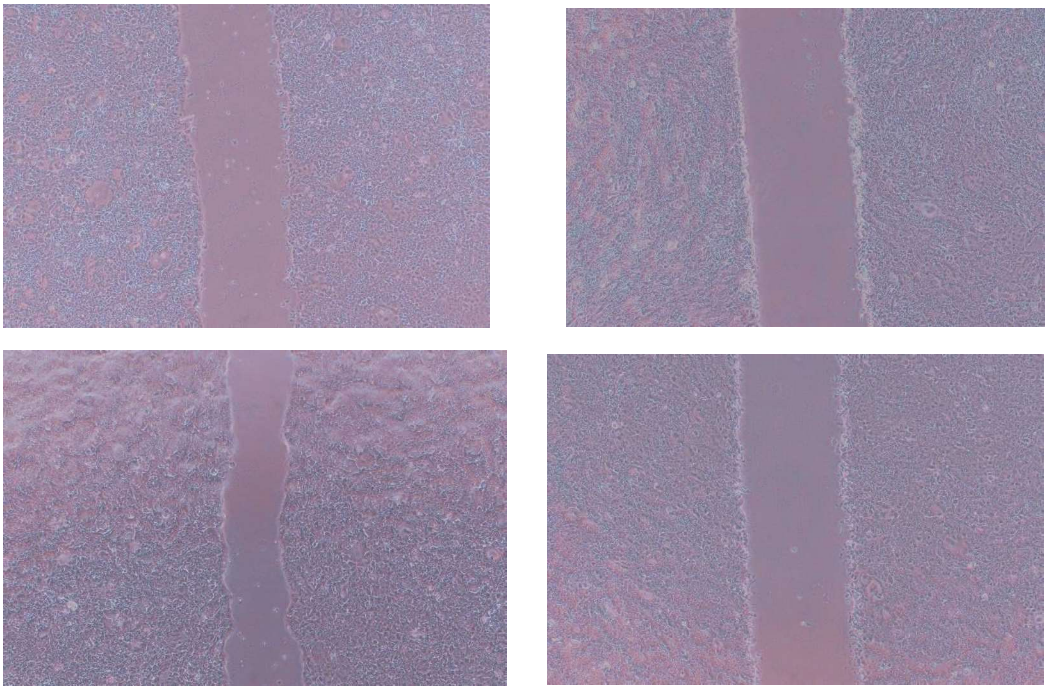

Related to Figure 8G

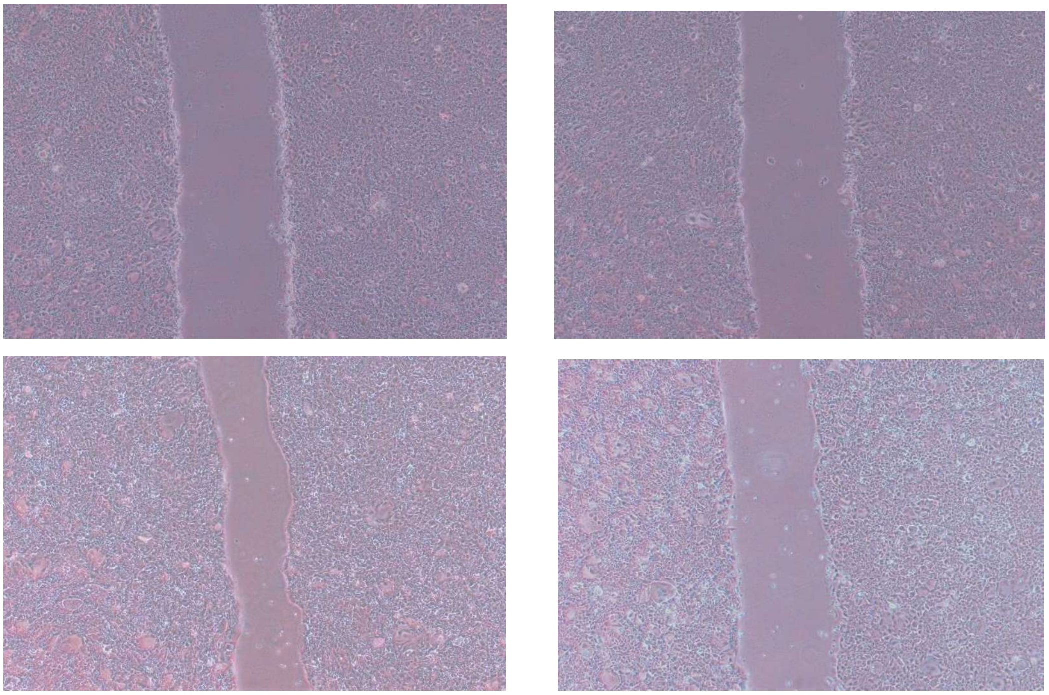

Original Images– Related to Figure 7H

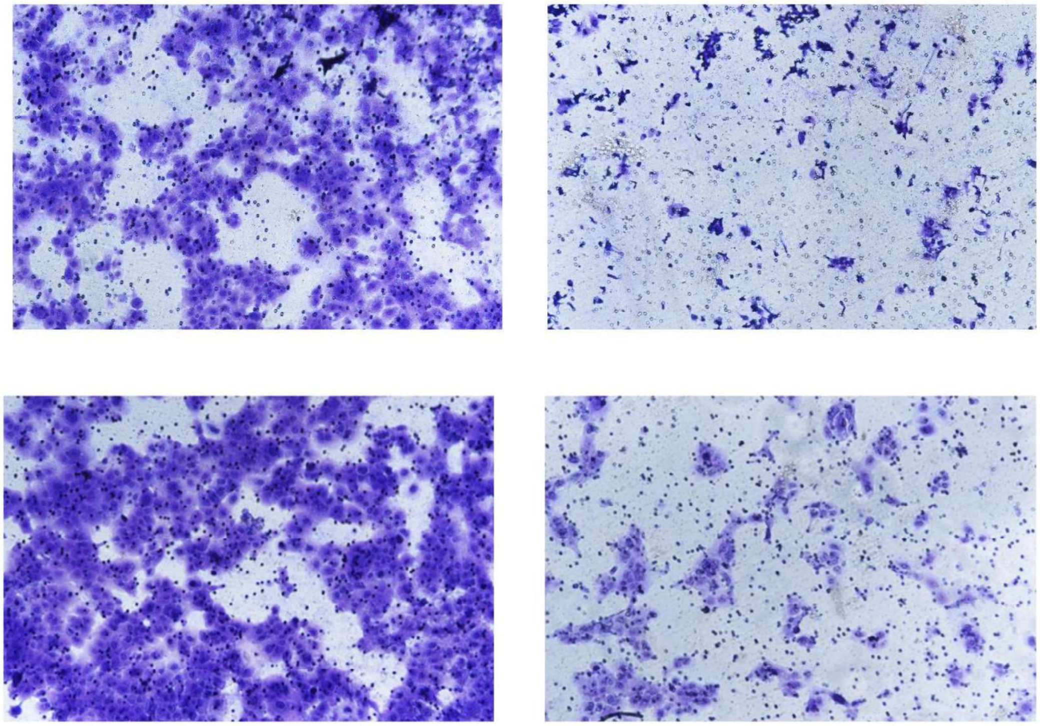

Related to Figure 8B

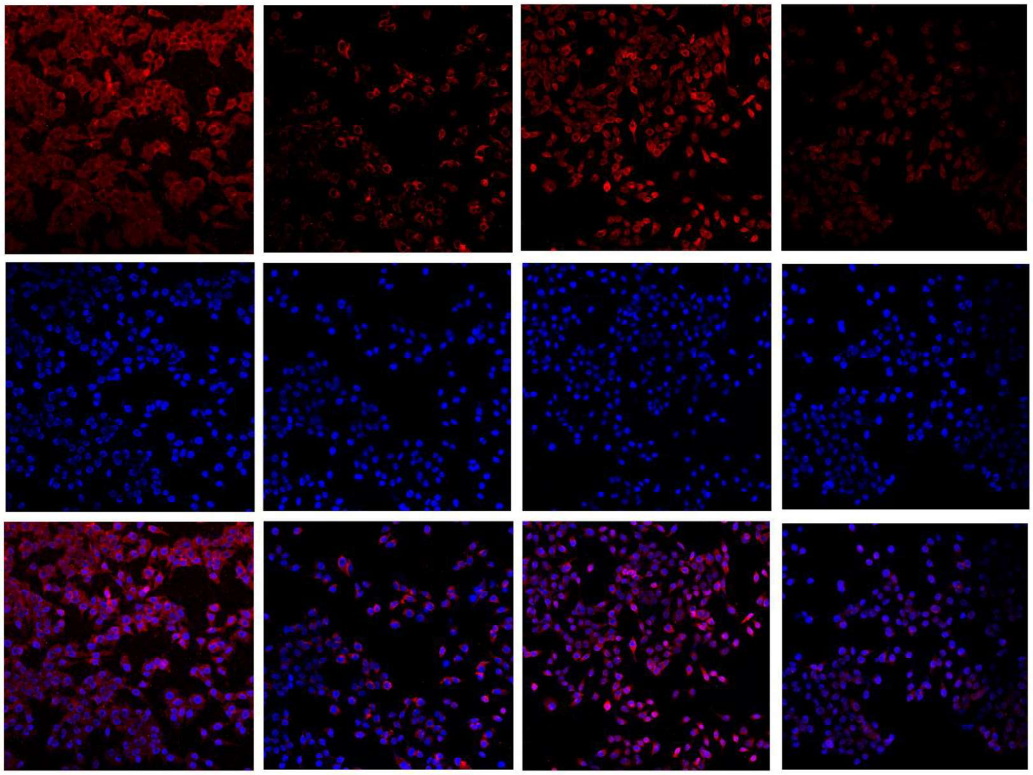

Related to Figure 8C

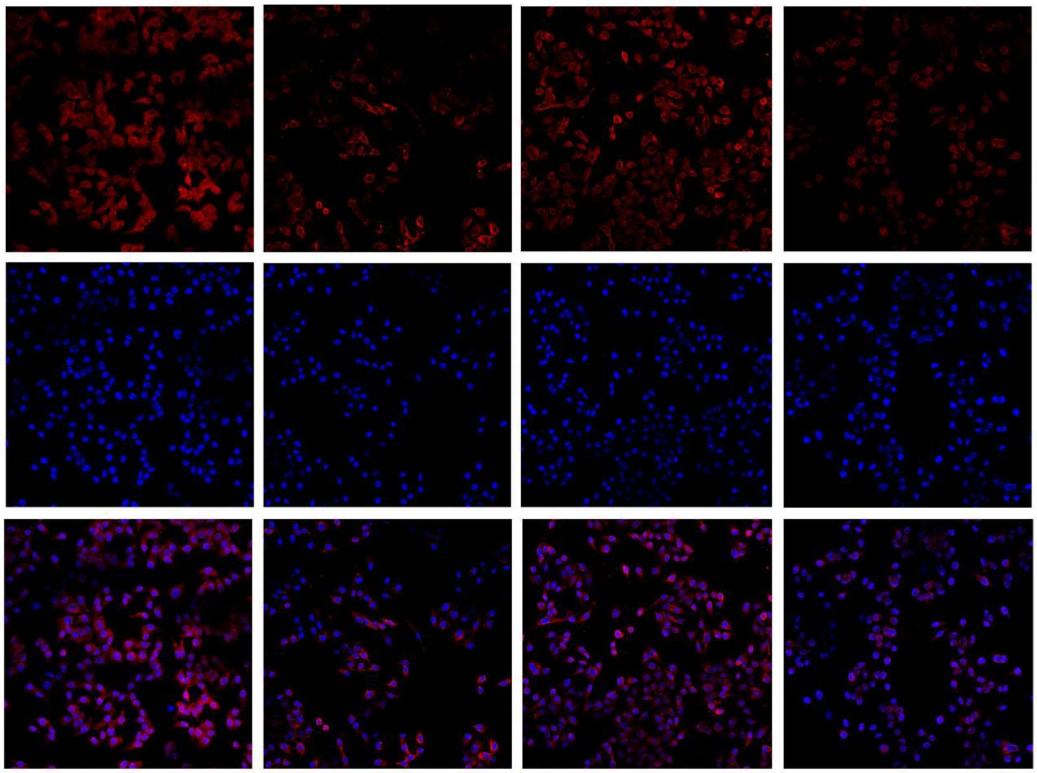

Related to Figure 8D

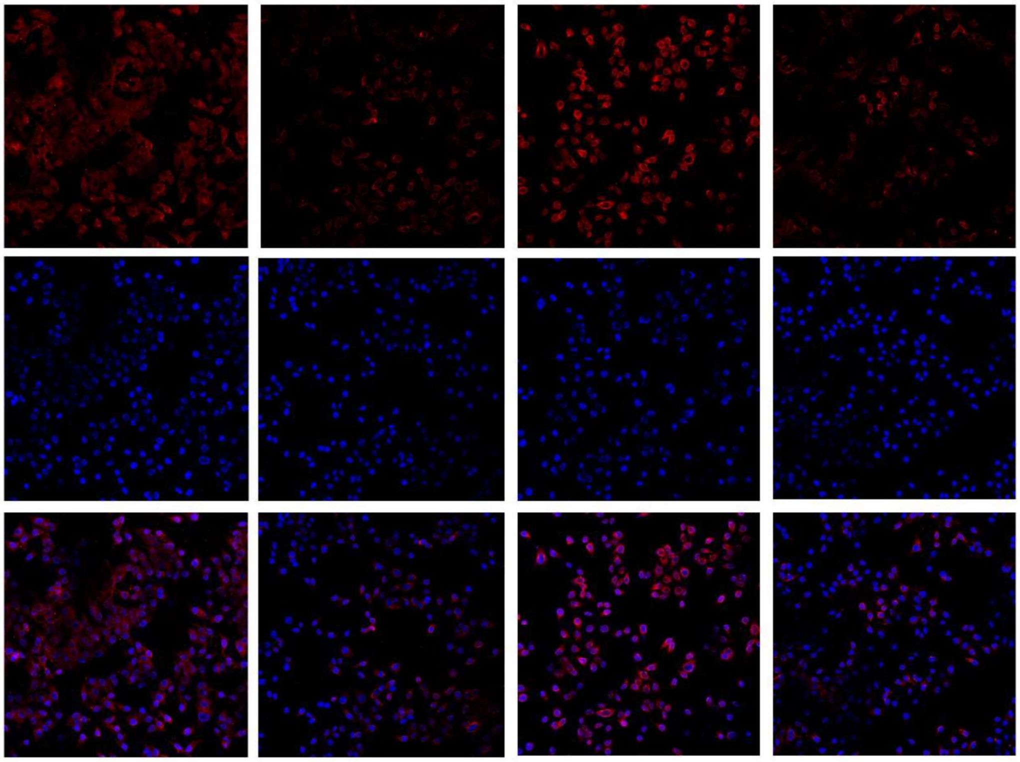

Supplement: Supplementary file 2 — Supplementary Material 2. [file 12957_2026_4299_MOESM2_ESM.pdf]
